# Effects of *Klebsiella michiganensis* LDS17 on *Codonopsis pilosula* growth, rhizosphere soil enzyme activities, and microflora, and genome-wide analysis of plant growth-promoting genes

**DOI:** 10.1128/spectrum.04056-23

**Published:** 2024-04-02

**Authors:** Tingting Jin, Jiahong Ren, Bianxia Bai, Wei Wu, Yongqing Cao, Jing Meng, Lihui Zhang

**Affiliations:** 1 Department of Life Sciences, Changzhi University, Changzhi, China; Dominican University New York, Orangeburg, New York, USA

**Keywords:** plant growth-promoting bacteria, ACC deaminase, *Klebsiella michiganensis*, plant growth-promoting property, soil enzyme activity, microbial functional diversity, whole-genome analysis, heavy metal resistance

## Abstract

*Codonopsis pilosula* is a perennial herbaceous liana with medicinal value. It is critical to promote *Codonopsis pilosula* growth through effective and sustainable methods, and the use of plant growth-promoting bacteria (PGPB) is a promising candidate. In this study, we isolated a PGPB, *Klebsiella michiganensis* LDS17, that produced a highly active 1-aminocyclopropane-1-carboxylate deaminase from the *Codonopsis pilosula* rhizosphere. The strain exhibited multiple plant growth-promoting properties. The antagonistic activity of strain LDS17 against eight phytopathogenic fungi was investigated, and the results showed that strain LDS17 had obvious antagonistic effects on *Rhizoctonia solani*, *Colletotrichum camelliae*, *Cytospora chrysosperma*, and *Phomopsis macrospore* with growth inhibition rates of 54.22%, 49.41%, 48.89%, and 41.11%, respectively. Inoculation of strain LDS17 not only significantly increased the growth of *Codonopsis pilosula* seedlings but also increased the invertase and urease activities, the number of culturable bacteria, actinomycetes, and fungi, as well as the functional diversity of microbial communities in the rhizosphere soil of the seedlings. Heavy metal (HM) resistance tests showed that LDS17 is resistant to copper, zinc, and nickel. Whole-genome analysis of strain LDS17 revealed the genes involved in IAA production, siderophore synthesis, nitrogen fixation, P solubilization, and HM resistance. We further identified a gene (*koyR*) encoding a plant-responsive LuxR solo in the LDS17 genome. *Klebsiella michiganensis* LDS17 may therefore be useful in microbial fertilizers for *Codonopsis pilosula*. The identification of genes related to plant growth and HM resistance provides an important foundation for future analyses of the molecular mechanisms underlying the plant growth promotion and HM resistance of LDS17.

**IMPORTANCE:**

We comprehensively evaluated the plant growth-promoting characteristics and heavy metal (HM) resistance ability of the LDS17 strain, as well as the effects of strain LDS17 inoculation on the *Codonopsis pilosula* seedling growth and the soil qualities in the *Codonopsis pilosula* rhizosphere. We conducted whole-genome analysis and identified lots of genes and gene clusters contributing to plant-beneficial functions and HM resistance, which is critical for further elucidating the plant growth-promoting mechanism of strain LDS17 and expanding its application in the development of plant growth-promoting agents used in the environment under HM stress.

## INTRODUCTION

*Codonopsis pilosula* is a perennial herbaceous liana belonging to the genus *Codonopsis* in the *Campanulaceae* family. *Codonopsis pilosula* roots can be used as medicines with various pharmacological effects (1). *Codonopsis pilosula* seedlings prefer moist, shady environments and are sensitive to bright light. Adverse stress commonly affects the normal growth of *Codonopsis pilosula*. At present, the growth ability of *Codonopsis pilosula* is improved primarily by increasing fertilizer application. However, long-term use of chemical fertilizers will accelerate heavy metal (HM) accumulation and soil acidification, resulting in soil compaction and nutrient imbalance. It is necessary to investigate safer and more environmentally friendly ways to improve the growth of *Codonopsis pilosula*.

Most soils contain a diverse range of bacteria, the ones that promote plant growth being referred to as plant growth-promoting bacteria (PGPB) (2). Given their environmental friendliness and sustainability, PGPB may be used to effectively replace or semi-replace chemical fertilizers, thereby alleviating the negative impacts on the soil environment caused by the widespread application of chemical fertilizers (3). The effects of PGPB on plant growth primarily comprise the following: (i) synthesizing and secreting plant auxin (4); (ii) transforming mineral elements in the soil from an unusable state to available form, thereby promoting the absorption of the mineral elements by plants (5, 6); (iii) nitrogen fixation (7); (iv) using siderophores to compete with pathogenic microorganisms to capture limited irons in the surrounding environment and inhibit the growth of pathogenic microorganisms (8); (v) inducing plant disease resistance (9); (vi) suppressing phytopathogenic fungi growth by secreting antibiotics (9); and (vii) reducing the ethylene concentration in plants by 1-aminocyclopropane-1-carboxylate (ACC) deaminase (10). Some PGPB exhibit HM resistance, which can improve the growth of plants in HM-stressed environments (11). PGPB typically exhibit one or more of the aforementioned plant growth-promoting (PGP) characteristics. When studying PGPB, researchers generally isolate and screen PGPB with a specific PGP property using a corresponding screening medium and then confirm the other PGP characteristics of the strain being assessed.

Glick (3) proposed that ACC deaminase is a key feature of PGPB in promoting plant growth. When plants are exposed to various environmental stressors (both biological and abiotic) during growth, ethylene is synthesized to activate their defense and protection systems (12, 13). When environmental stressors remain severe, plants produce excessive amounts of ethylene, which hinders plant growth and development by inhibiting root and stem proliferation and accelerating leaf aging (10). Many PGPB containing ACC deaminases have been used as inoculants. ACC deaminases produced by these PGPB can break down ethylene’s precursor ACC into α-ketobutyrate and ammonia, thereby reducing the ethylene concentration and preventing plant growth obstruction caused by excessive ethylene (14). Many researches have shown that ACC deaminase PGPB not only improve plant tolerance in stressful environments but also improve plant growth in normal environments (15–18).

PGPB can increase soil enzyme activity and modify the microbial community structure in plant rhizosphere soils, thereby indirectly promoting plant growth and environmental adaptability (19). For example, inoculation with the P-solubilizing bacteria *Pseudomonas fluorescens* CLW17 and *Bacillus cereus* CLY07 has been found to significantly increase alkaline phosphatase, invertase, and dehydrogenase activities in the *Taxus chinensis* var. *mairei* rhizosphere (20). Inoculation with four *Bacillus* strains was found to increase the number of cultivable bacteria and fungi in barley rhizosphere soil (21). The microbial community diversity in soil has further been found to be positively affected after the combined inoculation of four bacterial strains on *Allium cepa* L. seeds, with the Shannon-Wiener H′ and Chao-1 diversity indices being significantly increased compared to those of the control group (22).

Considering the PGP properties and environmental friendliness of PGPB, using PGPB may be an effective and sustainable way to improve the growth of *Codonopsis pilosula*. In this study, we isolated PGPB with ACC deaminase from the *Codonopsis pilosula* rhizosphere and screened for a PGPB strain, LDS17, that had high ACC deaminase activity. We used rhizosphere inoculation to confirm its effects on *Codonopsis pilosula* growth and rhizosphere soil qualities. We further sequenced and analyzed its whole genome, focusing on the genes involved in plant growth and HM resistance.

## MATERIALS AND METHODS

### Bacterial isolation, and ACC deaminase activity determination

The rhizosphere soils of *Codonopsis pilosula* plants were collected from four drought areas in the Shanxi province of China (Table S1) and used to isolate ACC deaminase-producing bacteria. ACC deaminase-producing bacteria were isolated from rhizospheric soils according to the procedure described by Penrose and Glick (23) with some modifications. Ten grams of soil samples was added into 90 mL of tryptic soy broth (TSB) media (23) in a 500-mL Erlenmeyer flask and shaken at 180 rpm for 24 h at 30°C. The incubation temperature of the isolates in the subsequent experiments was 30°C unless otherwise indicated. A 1-mL aliquot was removed from the growth culture, transferred to 50 mL of TSB media in a 250-mL Erlenmeyer flask, and incubated with shaking under the same conditions. After 24 h, 1 mL of the culture was added to 50 mL of DF minimal media (23) in a 250-mL Erlenmeyer flask. After incubation for 24 h (180 rpm), a 1-mL aliquot from each flask was transferred to 50 mL of DF minimal media with 3 mM ACC replacing (NH_4_)_2_SO_4_ as the sole nitrogen source (DFa) and incubated for 24 h (180 rpm). Finally, dilutions of the cultures were spread onto DFa minimal mediaagar and incubated for 24–48 h. Bacterial colonies were purified and maintained in 20% glycerol at −80°C for further use.

The ACC deaminase activity of each isolate was measured as described by Penrose and Glick (23). The activity of ACC deaminase was represented by the number of micromoles of α-ketobutyrate produced through ACC cleavage.

### Strain identification

The physiological and biochemical characteristics of the bacterial isolates were examined according to “Bergey’s Manual of Determinative Bacteriology” (24). The morphology of LDS17 was observed using a scanning electron microscope (Hitachi, TM3000). The 16S rRNA gene sequence of LDS17 was cloned according to the method described by Jin et al. (25) and compared with the 16S rRNA gene sequences of the type strains in the EzTaxon-e database. The 16S rRNA gene sequences of the type strains that matched the LDS17 16S rRNA gene sequence out of the top 24 results were used to build a neighbor-joining phylogenetic tree in MEGA 11.

### Plant growth promotion characteristics analysis

The IAA production and P solubilization ability were measured using the Salkowski colorimetric technique and the colorimetric molybdate blue method, respectively. For detailed operating methods, refer to the description of Jin et al. (26). Siderophore production was assessed with the method described by Schwyn and Neilands (27). The nitrogen-fixing ability of LDS17 was tested using N-free Ashby media (28). HCN and ammonia production were assessed according to the method described by Ahmad et al. (29). Antagonistic tests against eight pathogenic fungi (*Rhizoctonia solani*, *Colletotrichum camelliae*, *Cytospora chrysosperma*, *Phomopsis macrospore*, *Colletotrichum gloeosporioides*, *Fusicoccum aesculi*, *Rhizoctonia* sp., and *Botryosphaeria dothidea*) were performed. The details for antagonistic tests were described in the supplemental material.

### Preparation of bacterial inoculants

The test bacteria were incubated in 50 mL of TSB media with shaking at 180 rpm for 24 h. The bacterial cultures were then centrifuged at 12,000 × *g* and 4°C for 10 min. The cell pellets were transferred to 50 mL of DFa media and incubated for 24 h at 180 rpm. The bacterial cultures were then centrifuged again, washed thrice with sterile water, and resuspended in sterile water to prepare a 10^8^ CFU/mL inoculant.

### Pot culture of *Codonopsis pilosula* seedlings

*Codonopsis pilosula* seeds were surface sterilized with 75% ethanol for 30 s and 0.2% HgCl_2_ for 2 min and then rinsed with sterile water three times. Sterile seeds with uniform sizes were sown in plastic pots (5.0 cm in diameter and 7.5 cm in height) filled with 200 g of sterilized soil and unsterilized soil, respectively (organic carbon, 28.96 g/kg; available N, 35.0 mg/kg; available K, 25.4 mg/kg; and available P, 7.0 mg/kg). The pots were then placed in a greenhouse at 25°C (14 h in light and 10 h in the dark). The seedlings were thinned to five per pot after germination. One month after sowing, the bacterial suspensions (10^8^ CFU/mL) were inoculated into the rhizosphere of the *Codonopsis pilosula* seedlings, and an equal volume of sterile water was used as a blank control. The inoculum dose was 5 mL per seedling. Thirty replicates were used for each treatment group. On days 30, 60, and 90 after inoculation, the seedlings planted in the sterilized soil and their rhizosphere soil were collected to measure the seedling heights, ground diameters, biomasses, chlorophyll contents, and soil enzyme activity. The rhizosphere of the seedlings planted in unsterilized soil was used to determine the number of culturable microorganisms and the functional diversity of microbial communities.

### Determination of the total chlorophyll content

Fresh leaves (0.05 g) were ground in liquid nitrogen, after which 95% ethanol (2.5 mL) was added. The mixture was then incubated in the dark for 24 h. The absorbance values at 665 and 649 nm were determined using the resulting extracts. The total chlorophyll content was calculated using the following formula: total chlorophyll content = 6.63A_665_ + 18.08A_649_.

### Determination of soil enzyme activity

Invertase activity was determined using the method described by Frankenberger and Johanson (30). To assess the urease activity, 5 g of the soil samples and 1 mL of toluene were mixed in an Erlenmeyer flask. After 15 min, 10 mL of 10% urea solution and 20 mL of citrate buffer (citric acid 184 g/L and KOH 147.5 g/L, pH 6.7) were added to the flask. The flask was placed at 37°C for 24 h. Three milliliters of the filtrate was used to determine the NH_3_-N content through phenol-sodium hypochlorite colorimetry. Urease activity was measured in milligrams of NH_3_-N produced per gram of soil over 24 h. Catalase activity was determined using the method described by Johnson and Temple (31).

### Determination of culturable microorganisms

One gram of rhizosphere soil was added to 9 mL of sterile water and shaken vigorously for 20 min. The soil suspension was 10-fold gradient diluted, and each dilution of the series was spread onto beef extract peptone solid media, Gauze’s medium No. 1 agar, and Czapek-Dox Agar, respectively. The agar plates were incubated at 28°C until obvious colonies were formed. The colonies growing on beef extract peptone solid media, Gauze’s medium No. 1 agar, and Czapek-Dox Agar were used to calculate the CFU of bacteria, actinomycetes, and fungi, respectively. The number of culturable microorganisms was expressed as CFU per gram of rhizosphere soil.

### Determination of the functional diversity of microbial communities

One gram of rhizosphere soil was added to 9 mL of sterile water and shaken vigorously for 20 min. The supernatant was diluted 100 times before being transferred to Biolog Eco MicroPlates (100 µL per well). The Biolog Eco MicroPlates were incubated at 25°C for 7 days. The absorbance values at 590 and 750 nm were measured every 24 h. The absorbance at 590 nm minus that at 750 nm was used as the absorbance value for the reaction. The average well color development (AWCD) represented microbial activity. The Shannon, Simpson, and McIntosh indices were used to represent microbial carbon source utilization. The AWCD, Shannon, Simpson, and McIntosh indices were calculated using the formulae described by Ge et al. (32) and Manjunath et al. (33).

### Whole-genome sequencing and annotation

The whole-genome sequencing of strain LDS17 was performed by Hangzhou Woosen Biotechnology Co., Ltd., China. In brief, a genomic DNA library was constructed using the MagicSeq DNA Library Prep Plus Kit from Illumina (MagicBio, M316). The NovaSeq 6000 platform was used for sequencing. Subread error correction was performed using Canu v1.5, followed by contig construction using miniasm (0.2-r159). Pilon v1.22 software was used to correct the contigs. Gene annotation and functional predictions were performed using NCBI and Integrated Microbial Genomes & Microbiomes (IMG, https://img.jgi.doe.gov/).

### Comparative genomics analysis

We extracted the whole-genome sequences of the model strains (using the genome published in GenBank) that ranked among the top 23 in terms of their similarity to the LDS17 16S rRNA gene sequence in the EzTaxon-e database and compared the average nucleotide identity (ANI) with LDS17. We substituted the reference genome of the same species to the GenBank database for the model strain, the full genome information of which has not yet been published. The online ANI calculator (http://enve-omics.ce.gatech.edu/ani/) was used to calculate the ANI of the whole-genome sequences of LDS17 and other strains. MEGA 11 software was used to construct a phylogenetic tree based on the nucleotide sequences of six housekeeping genes (*recA*, *gyrB*, *rpoA*, *rpoB*, *rpoC*, and *rpoD*).

### Analysis of genes related to plant growth and HM resistance

IAA synthesis pathways were predicted by screening genes related to indole-3-acetate biosynthesis and tryptophan metabolism in the IMG platform. AntiSMASH was used to identify siderophore synthetic gene clusters. Genes involved in ACC deaminase production, P solubilization, nitrogen fixation, as well as HM resistance (copper, arsenic, nickel, cobalt, and zinc) were identified according to the gene functional annotations performed by NCBI and IMG. Pairwise identity percentages between amino acid sequences of identified PGP and HM resistance-related proteins and known corresponding proteins were calculated using the European Molecular Biology Laboratory-European Bioinformatics Institute European Molecular Biology Open Software Suite Needle tool.

### HM resistance experiment

The overnight bacterial culture of LDS17 was diluted to an OD_600_ value of 0.5 before being inoculated into fresh LB media (5%, vol/vol) containing various concentrations of HM salts. The concentrations of HM salts in the LB media were as follows: CuSO_4_: 2, 4, and 5 mM; ZnSO_4_: 1 and 2 mM; and NiCl_2_: 1 and 2 mM. The bacterial culture was transferred to a 48-well plate (600 µL per well). No bacterial cultures were added to the outermost wells of the 48-well culture plate, and only an equal volume of liquid LB media was added. The 48-well culture plate was placed in a plate reader (BioTek, Synergy H1) for an oscillating culture at 30°C, after which the growth curve was measured. The parameters were as follows: shaking mode, double orbital (continuous); shaking frequency, 365 cpm (2 mm); and reading wavelength, 600 nm. The data were read every 15 min.

## RESULTS

### Isolation and screening of ACC deaminase-producing bacteria

A total of 24 isolates with ACC deaminase activity were obtained from the rhizosphere soil of *Codonopsis pilosula* collected from four sampling sites. The ACC deaminase activities produced by these 24 isolates ranged from 0.18 to 5.29 µmol α-ketobutyrate/mg protein/min. The LDS17 strain exhibited the highest ACC deaminase activity (5.29 µmol α-ketobutyrate/mg protein/min) and was therefore selected for further analysis (Fig. S1).

**Fig 1:**
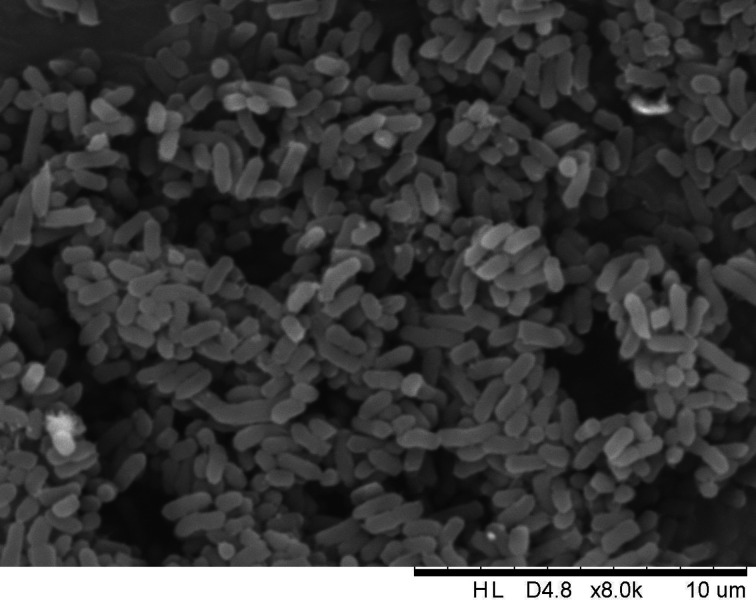
Scanning electron microscope photo of *Klebsiella michiganensis* LDS17 isolated from *Codonopsis pilosula* rhizosphere.

LDS17 cells are Gram-negative and short rod-shaped, with diameters and lengths of 0.5–0.8 µm and 1–2 µm, respectively (Fig. 1). The 16S rRNA gene sequence of LDS17 was 99.63% similar to that of *Klebsiella michiganensis* W14^T^. The phylogenetic tree based on 16S rRNA gene sequences (Fig. S2) showed that LDS17 and *Klebsiella michiganensis* W14^T^ were clustered in a single subclade. These results suggest that LDS17 belongs to the *Klebsiella* genus.

### Plant growth promotion characteristics and antagonistic activity against the phytopathogenic fungi of strain LDS17

**Fig 2:**
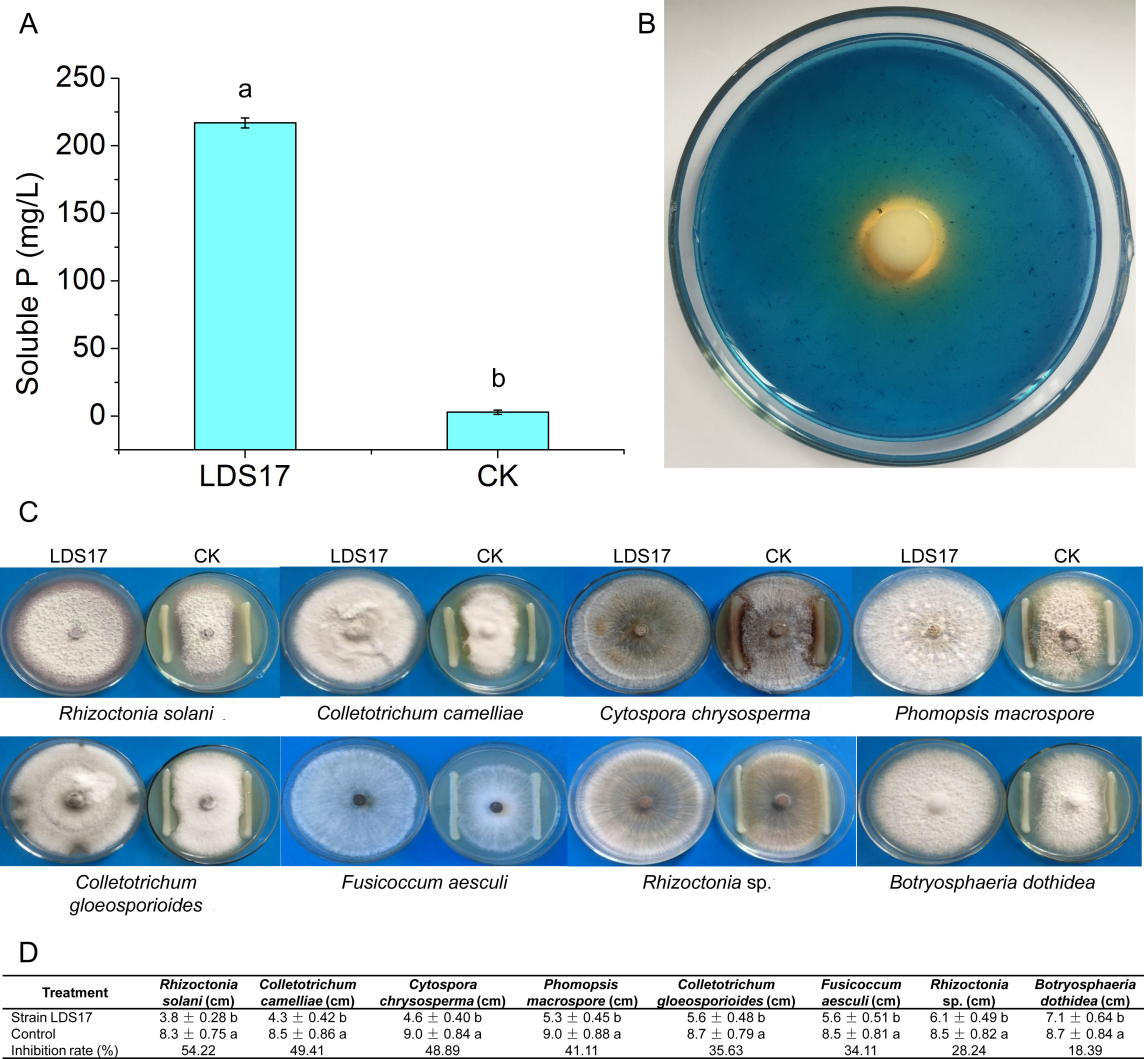
The abilities of *Klebsiella michiganensis* LDS17 to dissolve insoluble P by determining the soluble P concentration in the culture supernatant of LDS17 (**A**), produce siderophore on the CAS agar plate (**B**), and inhibit plant pathogenic fungi (**C and D**). (A) Different letters in the column indicate significant differences (*P* < 0.05). (D) Values are means ± SD, different letters indicate significant differences (*P* < 0.05).

IAA (8.77 mg/L) was produced in the supernatant of the LDS17 culture after 7 days of incubation. On day 6 of the incubation period, the concentration of soluble P in the culture supernatant was 216.89 mg/L (Fig. 2A). A chelated halo was observed around the LDS17 colony on the CAS agar plate (Fig. 2B), indicating the siderophore-producing ability of LDS17. LDS17 was able to produce HCN and ammonia and grew in N-free Ashby media. Therefore, the LDS17 strain has many PGP properties. We also tested the antagonistic activity of LDS17 against eight plant pathogenic fungi (Fig. 2C). The growth inhibition rates of LDS17 for these eight pathogenic fungi were as follows: *Rhizoctonia solani*, 54.22%; *Colletotrichum camelliae*, 49.41%; *Cytospora chrysosperma*, 48.89%; *Phomopsis macrospore*, 41.11%; *Colletotrichum gloeosporioides*, 35.63%; *Fusicoccum aesculi,* 34.11%; *Rhizoctonia* sp., 28.24%; and *Botryosphaeria dothidea*, 18.39% (Fig. 2D). The results indicated that LDS17 possessed varying degrees of antagonistic activity against these eight pathogenic fungi, with obvious antagonistic effects on *Rhizoctonia solani*, *Colletotrichum camelliae*, *Cytospora chrysosperma*, and *Phomopsis macrospore*.

### LDS17 inoculation promotes the growth of *Codonopsis pilosula* seedlings

**Fig 3:**
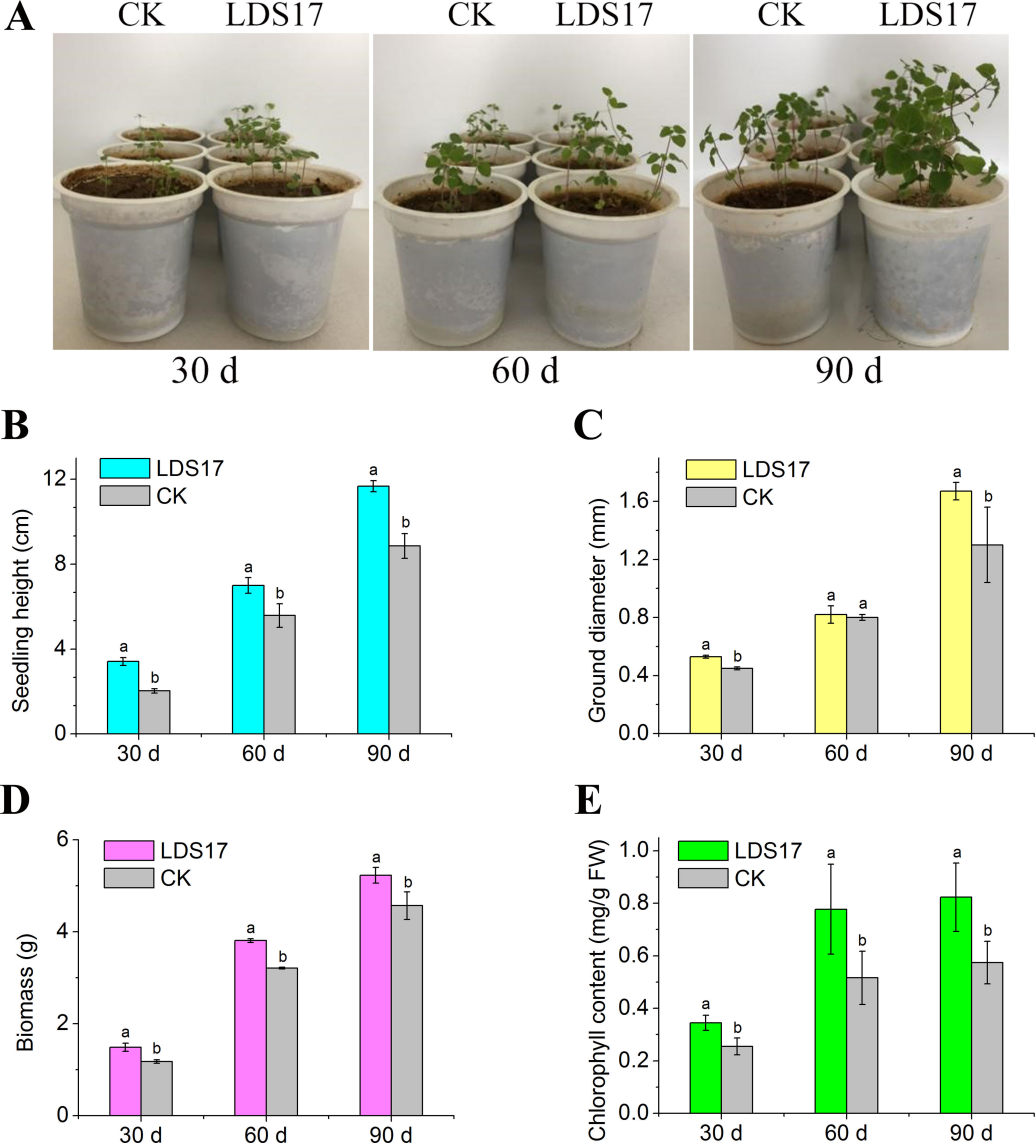
Inoculation effects of *Klebsiella michiganensis* LDS17 on *Codonopsis pilosula* seedlings. (A) Growth of *Codonopsis pilosula* seedlings after *Klebsiella michiganensis* LDS17 inoculation. (B–E) Seedling height, ground diameter, biomass, and chlorophyll content of *Codonopsis pilosula* seedlings after *Klebsiella michiganensis* LDS17 inoculation. Different letters in the column indicate significant differences (*P* < 0.05).

The inoculation of LDS17 significantly promoted the growth of *Codonopsis pilosula* seedlings (Fig. 3). On day 30 after the inoculation, the heights, ground diameters, and biomasses of the *Codonopsis pilosula* seedlings increased by 67.98%, 17.78%, and 26.27%, respectively. By day 60 after the inoculation, the seedling diameters had not grown significantly; however, their heights and biomasses had increased by 25.45% and 18.69%, respectively. On day 90 after the inoculation, the heights, ground diameters, and biomasses of the *Codonopsis pilosula* seedlings increased by 31.72%, 28.46%, and 14.44%, respectively (Fig. 3B, C, and D). These results suggest that LDS17 can continuously promote the growth of *Codonopsis pilosula* seedlings within 90 days of inoculation. Additionally, chlorophyll content measurements revealed that the inoculation of LDS17 was beneficial for the accumulation of total chlorophyll; on days 30, 60, and 90 after the inoculation, the total chlorophyll content of the *Codonopsis pilosula* seedlings increased by 35.29%, 50.58%, and 43.38%, respectively (Fig. 3E).

### LDS17 inoculation improves soil enzyme activity in the rhizosphere soil of *Codonopsis pilosula* seedlings

**Fig 4:**
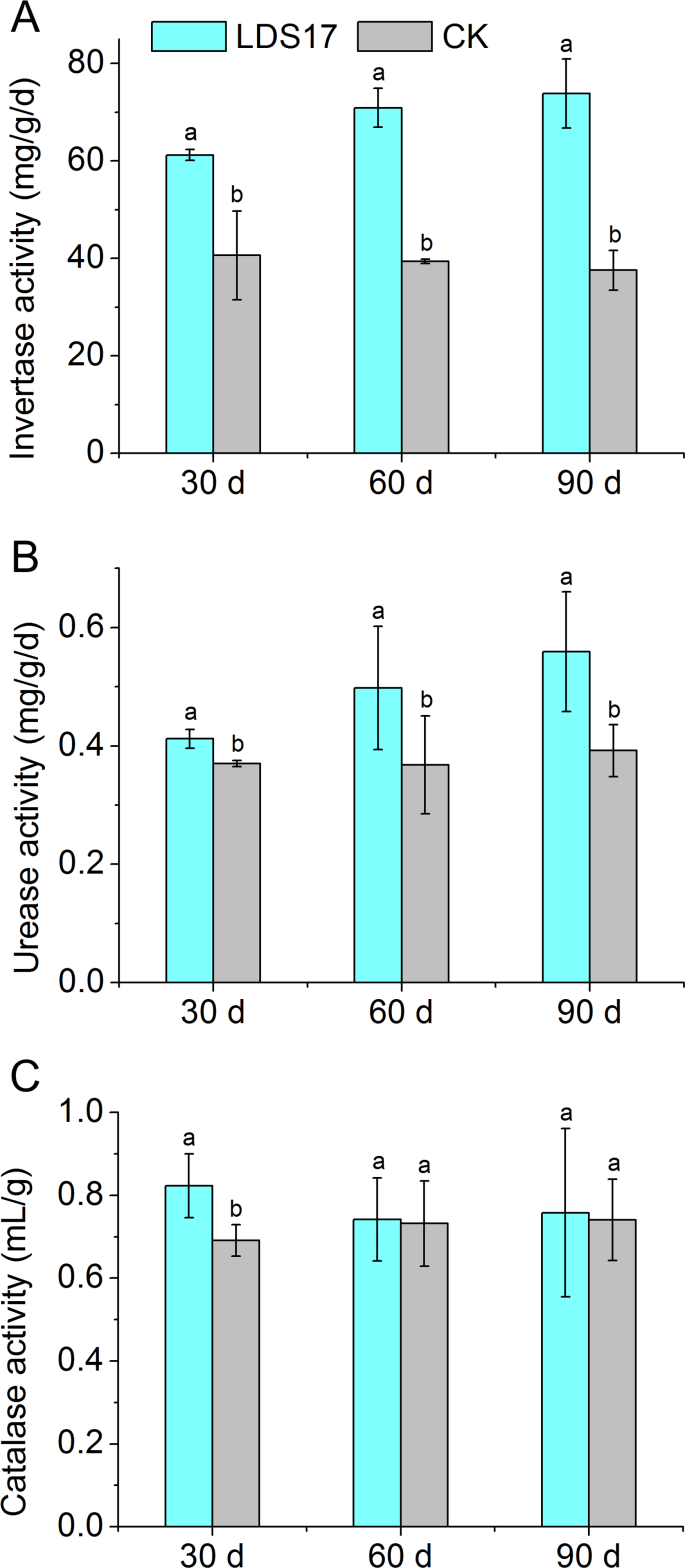
Soil enzyme activities in *Codonopsis pilosula* rhizosphere soil after *Klebsiella michiganensis* LDS17 inoculation. (A) Invertase activity. (B) Urease activity. (C) Catalase activity. Different letters in the column indicate significant differences (*P* < 0.05).

We tested the effects of LDS17 inoculation on the invertase, urease, and catalase activities in the rhizosphere soil of *Codonopsis pilosula* seedlings. The activities of invertase and urease in the rhizosphere of the *Codonopsis pilosula* seedlings gradually increased after the LDS17 inoculation and were significantly greater than those in the control group. The invertase activity in the rhizosphere soil of the seedlings was 1.51-, 1.80-, and 1.97-times that of the control group on days 30, 60, and 90 after the LDS17 inoculation, respectively (Fig. 4A), and the urease activity was 1.11-, 1.35-, and 1.43-times that of the control group (Fig. 4B). However, the effects of the LDS17 inoculation on the catalase activity in the rhizosphere soil of the *Codonopsis pilosula* seedlings were not significant and were only slightly higher than that of the control group (1.19 times) on day 30 after the inoculation. There were no significant differences in the catalase activity in the rhizosphere soil on days 60 and 90 after the inoculation compared with that in the control group (Fig. 4C). These results indicate that the inoculation of LDS17 led to a continuous increase in the invertase and urease activities in the *Codonopsis pilosula* rhizosphere soil.

### Effects of LDS17 inoculation on culturable microorganisms in the rhizosphere of *Codonopsis pilosula* seedlings

**Fig 5:**
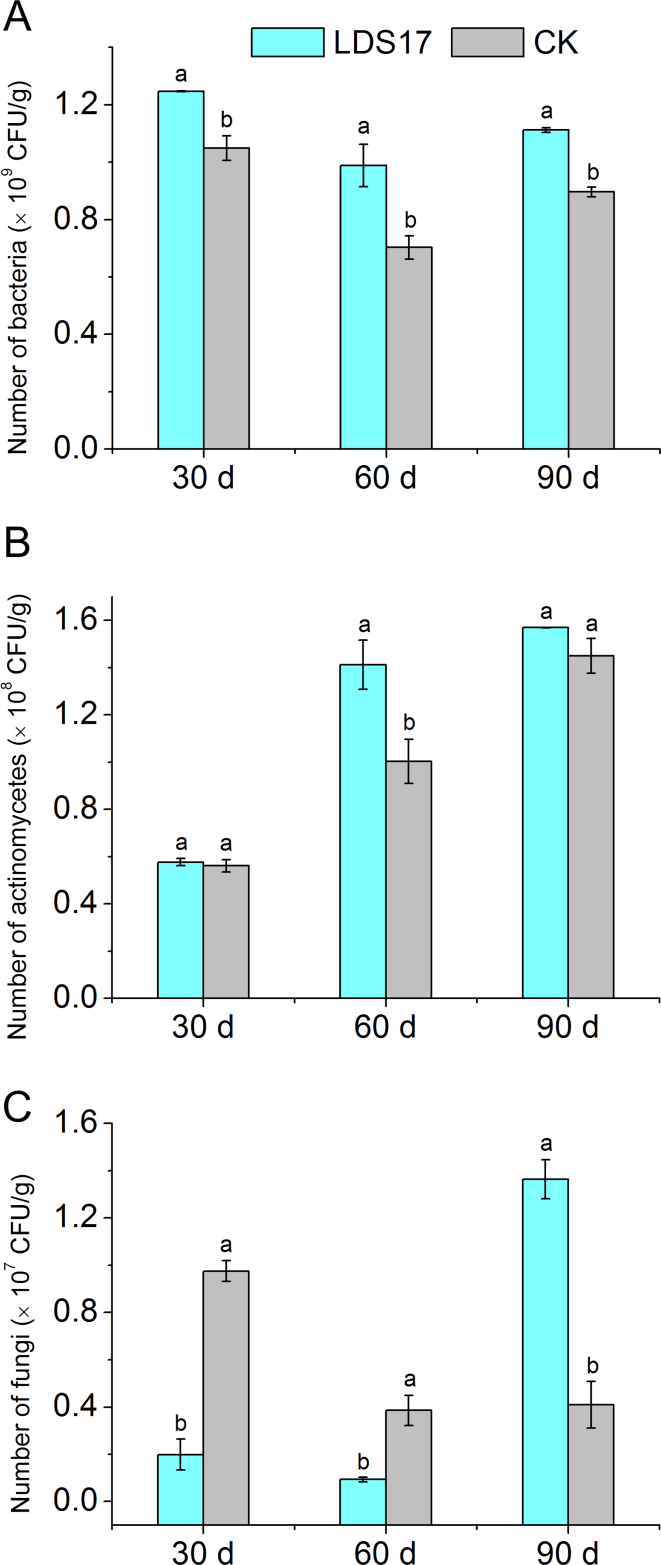
Culturable microorganism numbers in *Codonopsis pilosula* rhizosphere soil after *Klebsiella michiganensis* LDS17 inoculation. (A) Culturable bacteria. (B) Culturable actinomycetes. (C) Culturable fungi. Different letters in the column indicate significant differences (*P* < 0.05).

On days 30, 60, and 90 after the LDS17 inoculation in the rhizosphere of the *Codonopsis pilosula* seedlings, the number of culturable bacteria was significantly higher (1.19-, 1.41-, and 1.24-times, respectively) than that in the control group (Fig. 5A). The abundance of actinomycetes in the rhizosphere of the *Codonopsis pilosula* seedlings on day 30 after the LDS17 inoculation did not differ from that in the control group. As the inoculation time increased, the number of actinomycetes in the rhizosphere soil of the seedlings gradually increased compared with that in the control group and reached 1.41 times that of the control group on day 60 after the LDS17 inoculation. The difference between the two groups gradually narrowed on day 90 after the LDS17 inoculation, and the abundance of actinomycetes in the inoculation treatment group was only 1.08 times that of the control group (Fig. 5B). The number of fungi in the rhizosphere soil of the *Codonopsis pilosula* seedlings was lower on days 30 and 60 after the LDS17 inoculation than in the control group but increased significantly on day 90, reaching 3.33 times that of the control group (Fig. 5C). The LDS17 inoculation, therefore increased the number of culturable microorganisms in the rhizosphere of the *Codonopsis pilosula* seedlings to a certain extent.

### Effects of LDS17 inoculation on the functional diversity of microbial communities in the rhizosphere of *Codonopsis pilosula* seedlings

Biolog Eco MicroPlates were used to evaluate the functional diversity of the microbial communities in the rhizosphere of the *Codonopsis pilosula* seedlings with LDS17 inoculation. The AWCD of the inoculation treatment group was not significantly different from that of the control group on days 30 and 60 after the LDS17 inoculation. The AWCD value of the inoculation treatment group gradually became higher than that of the control group on day 90 after inoculation as the incubation time of the soil samples was extended. This suggests that the inoculation of LDS17 improves the carbon source utilization and metabolic activity of microorganisms in the rhizosphere of *Codonopsis pilosula* seedlings (Fig. S3).

The Shannon index of the rhizosphere soil of the *Codonopsis pilosula* seedlings was lower than that of the control group on day 30 after the LDS17 inoculation and was the same as it had been on day 60 but significantly higher than that of the control group on day 90. The Simpson and McIntosh indices of the rhizosphere soil of the *Codonopsis pilosula* seedlings were significantly higher than those of the control group during the three time periods (30, 60, and 90 days) after the LDS17 inoculation (Table 1). These results indicate that the inoculation of *Codonopsis pilosula* seedlings with LDS17 improves the richness, dominance, and evenness of microbial species in the rhizosphere soil.

**TABLE 1:** Effects of *Klebsiella michiganensis* LDS17 inoculation on the functional diversity indices of microbial communities in rhizosphere soil of *Codonopsis pilosula^a^*

Inoculation time (days)	Treatment	Shannon index	Simpson index	McIntosh index
30	LDS17	3.009 ± 0.045 d	0.944 ± 0.001 b	7.647 ± 0.305 d
	CK	3.190 ± 0.067 c	0.910 ± 0.001 d	8.434 ± 0.135 c
60	LDS17	3.330 ± 0.122 b	0.992 ± 0.002 a	10.943 ± 0.188 a
	CK	3.251 ± 0.091 bc	0.919 ± 0.013 cd	9.707 ± 0.136 b
90	LDS17	3.436 ± 0.133 a	0.999 ± 0.012 a	9.398 ± 0.421 b
	CK	3.259 ± 0.022 bc	0.928 ± 0.013 c	7.906 ± 0.351 cd

^*a*^ Functional diversity indices are based on carbon source utilization patterns measured in BIOLOG Eco MicroPlates. CK, without LDS17 strain inoculation. Values are means ± SD; different letters indicate significant differences (*P* < 0.05).

### Analysis of the whole-genome characteristics of LDS17

**Fig 6:**
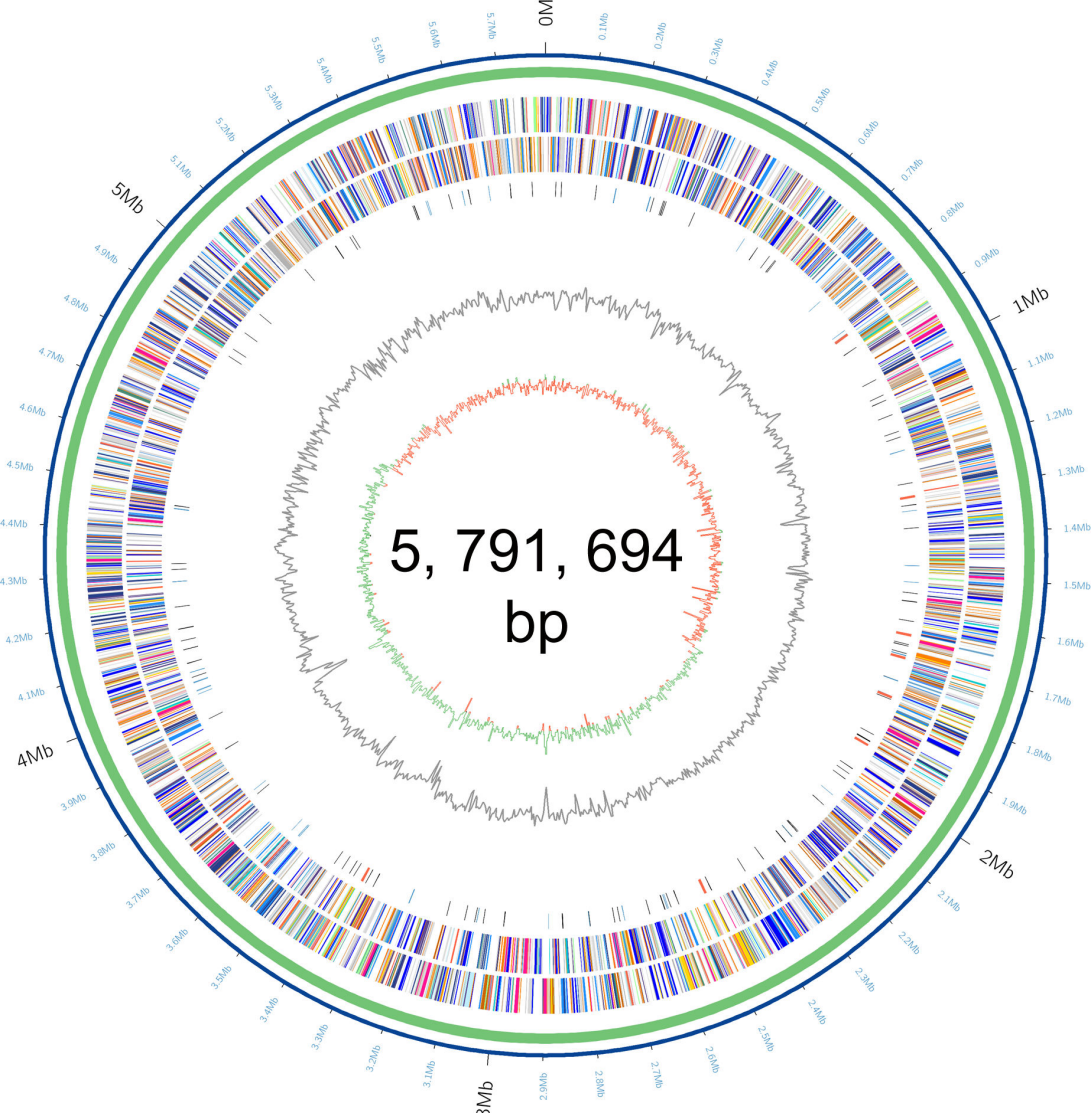
*Klebsiella michiganensis* LDS17 genome circle map of *Klebsiella michiganensis* LDS17. From outside to inside, the circle is represented as follows: circle 1: genome size; circles 2 and 3: gene distribution in positive and negative chains; circle 4: ncRNA (black), tRNA (blue), and rRNA (red); circle 5: GC ratio; and circle 6: GC skew.

The LDS17 genome contained one chromosome, with a size of 5,791,694 bp and a GC ratio of 56.12% (Fig. 6). The entire LDS17 genome contained 5,287 protein coding genes, 25 rRNAs, 84 tRNAs, and 89 pseudogenes.

### Phylogeny and comparative genome analysis of LDS17

**Fig 7:**
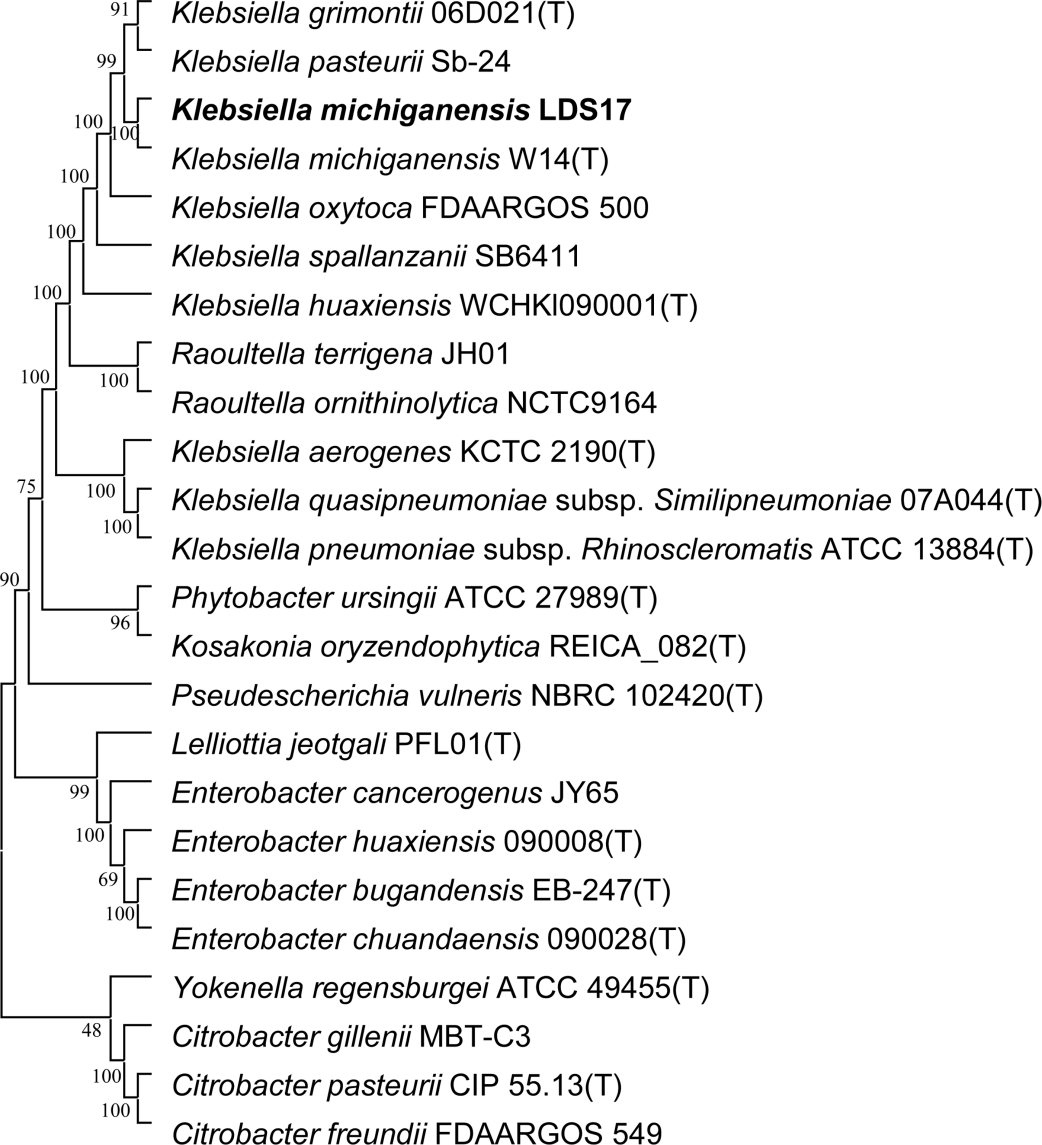
Phylogenetic tree based on housekeeping genes *recA*, *gyrB*, *rpoA*, *rpoB*, *rpoC*, and *rpoD* indicates the phylogenetic position of the strain LDS17 and its relationship with related species. The number on each node represents the bootstrap value (1,000 replications). The GenBank accession numbers of the housekeeping genes are shown in Table S3.

LDS17 and *Klebsiella michiganensis* W14^T^ had the highest ANI values (99.07%), exceeding the ANI classification threshold by 95% (Table S2). In the phylogenetic tree based on the nucleotide sequences of six housekeeping genes, LDS17 and *Klebsiella michiganensis* W14^T^ were clustered on the same branch (Fig. 7), which was consistent with the ANI comparison results. Therefore, we determined that LDS17 belongs to *Klebsiella michiganensis*.

### Analysis of the plant growth-related genes of LDS17

#### ACC deaminase

We discovered a potential ACC deaminase-coding gene, *I4W82_18900*, in the LDS17 genome, with 35.7% and 53% amino acid sequence identity and similarity to those of the ACC deaminase (AcdS, PputUW4_04154) in *Pseudomonas* sp. UW4 (34). Nascimento et al. (35) discovered that five amino acid residues (Lys51, Ser78, Tyr294, Glu295, and Leu322) in the *Pseudomonas* sp. UW4 AcdS sequence were required for ACC deaminase activity. The coding product of gene *I4W82_18900* in LDS17 contained three key amino acid residues: Lys51, Ser78, and Tyr294.

#### IAA

Four genes involved in two IAA synthetic pathways, IAN (indole-3-acetonitrile) and IPyA (indole-3-pyruvate) pathways, were identified in the LDS17 genome (Table S4). In the IAN pathway, IAN is converted to IAM (indole-3-acetamide) and then to IAA by the catalysis of nitrile hydratase (*I4W82_22605* and *I4W82_22610*) and amidase (I4W82_22600). Genes encoding enzymes that catalyze the conversion of tryptophan to IAN were not identified in the LDS17 genome. *I4W82_16760* coded for indolepyruvate decarboxylase, which converts IPyA to IAAld (indole-3-acetaldehyde). The gene encoding aldehyde dehydrogenase, which catalyzes the oxidation of IAAld to IAA, was absent in the LDS17 genome.

#### P solubilization

Many PGPB primarily use gluconic acid to convert insoluble P into a form that can be directly absorbed by plants (36). In the LDS17 genome, we discovered a gene encoding glucose dehydrogenase (*I4W82_04525*) (Table S4), which catalyzes the formation of gluconic acid from glucose. However, we did not identify genes encoding the cofactor pyrroloquinoline (PQQ) for glucose dehydrogenase (37).

#### Enterobactin

**Fig 8:**
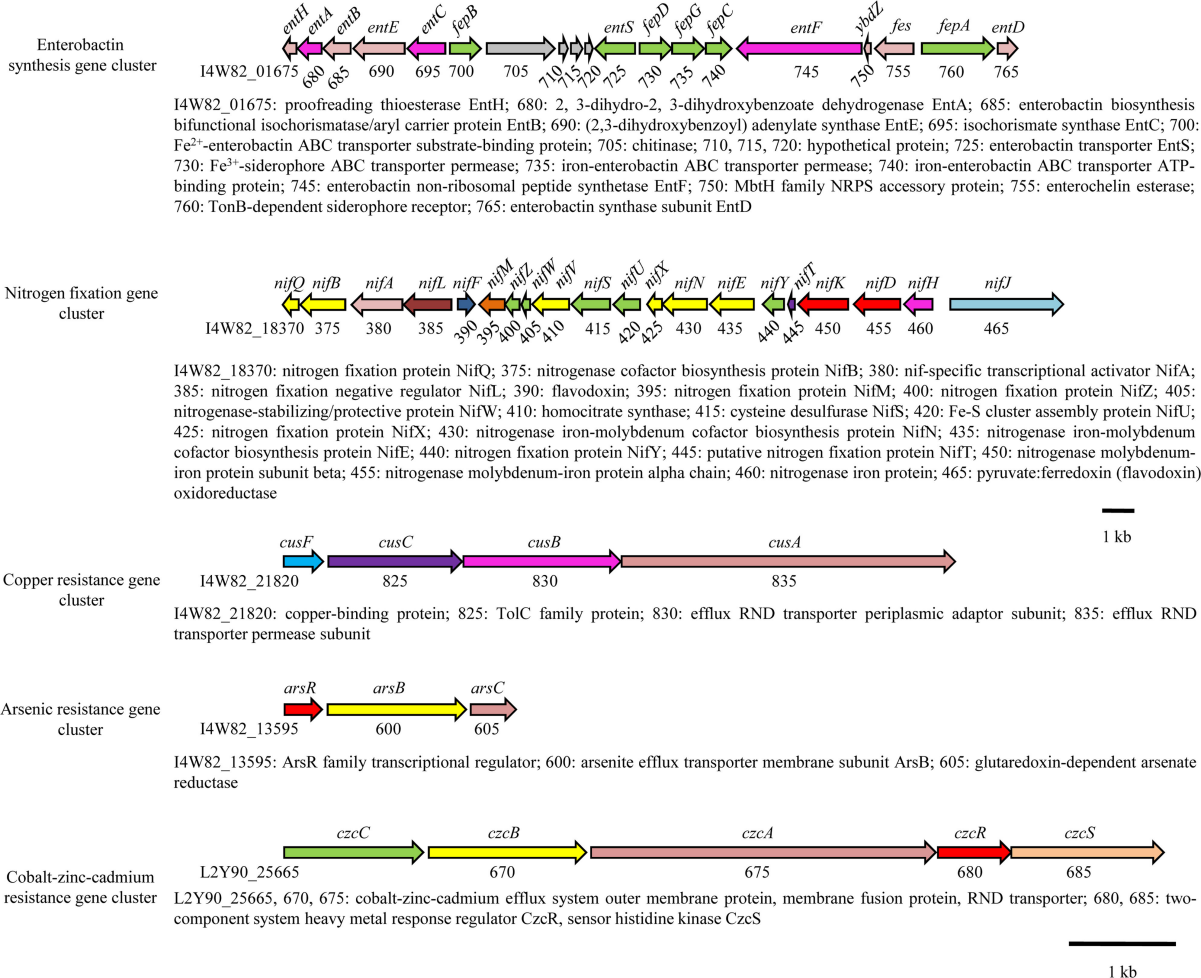
Gene clusters associated with plant growth promotion and HM resistance.

**Fig 9:**
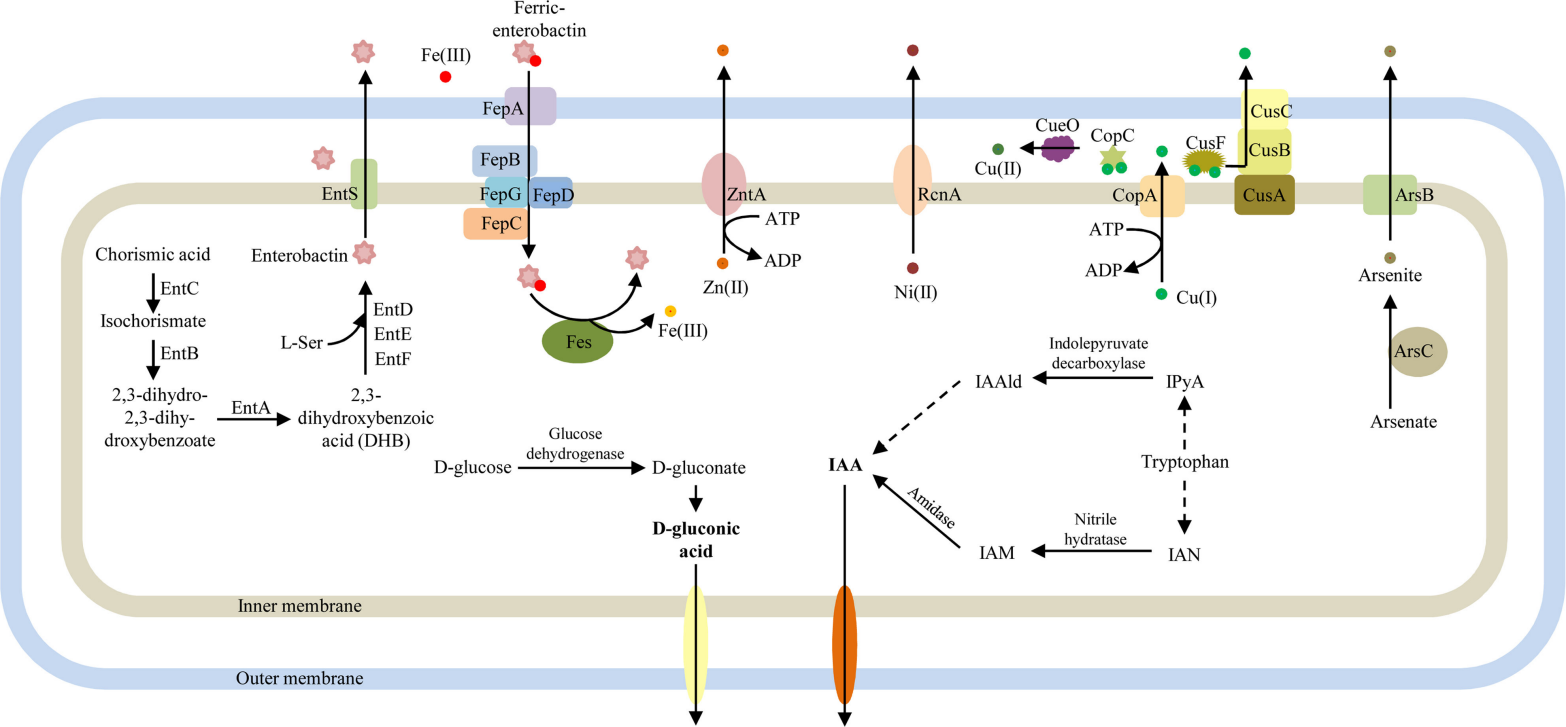
Speculative pattern diagram of plant growth promotion and HM resistance mechanisms in *Klebsiella michiganensis* LDS17.

A complete enterobactin (38) siderophore synthesis gene cluster was identified in the LDS17 genome. The length of the enterobactin gene cluster was 22,213 bp and composed of 19 genes (Table S4; Fig. 8 and 9): (i) eight genes related to enterobactin synthesis (*entA*, *entB*, *entC*, *entD*, *entE*, *entF*, *entH*, and *ybdZ*). The coding products of *entC* (*I4W82_01695*), *entB* (*I4W82_01685*), and *entA* (*I4W82_01680*) catalyzed the synthesis of 2,3-dihydroxybenzoate (DHB) from the chorismic acid precursor; the coding products of *entD* (*I4W82_01765*), *entE* (*I4W82_01690*), *entF* (*I4W82_01745*), and *entB* were responsible for the polymerization and cyclization of DHB and L-serine to form enterobactin (38); *entH* (*I4W82_01675*) encoded a thioesterase that optimized the biosynthesis of enterobatin by interacting with the ArCP domain of EntB (39); *ybdZ* (*I4W82_01750*) coded for the MbtH family protein, which was thought to improve the catalytic activity of EntF (40). (ii) Seven enterobactin transporter-related genes (*entS*, *fepA*, *fepB*, *fepC*, *fepD*, *fepG*, and *fes*). *EntS* (*I4W82_01725*) encoded a membrane transporter protein that transports enterobactin from the intracellular to the extracellular space (41). Enterobactin is recognized by a specific receptor encoded by *fepA* (*I4W82_01760*) after binding to iron and is transported to the intracellular space via the products encoded by *fepB* (*I4W82_01700*), *fepD* (*I4W82_01730*), *fepG* (*I4W82_01735*), and *fepC* (*I4W82_01740*). Ferric enterobactin is then catalyzed by enterobactin esterase encoded by *fes* (*I4W82_01755*), resulting in the release of iron into the cytoplasm.

#### Nitrogen fixation

A 23,296-bp *nif* gene cluster involved in nitrogen fixation was identified in the LDS17 genome (Table S4; Fig. 8). This gene cluster consisted of 20 genes, including genes related to MoFe protein (*nifD*, *nifK*, *nifB*, *nifQ*, *nifX*, *nifN*, *nifE*, *nifV*, *nifH*, *nifY*, *nifW*, *nifZ*, *nifS*, and *nifU*) and Fe protein biosynthesis (42, 43).

### Identification of HM resistance-related genes

Thirteen copper resistance genes were identified in the LDS17 genome (Table S4; Fig. 8). *CusF* (*I4W82_21820*), *cusC* (*I4W82_21825*), *cusB* (*I4W82_21830*), and *cusA* (*I4W82_21835*) are located in the same gene cluster, and their coding products were homologous to that of the periplasmic chaperone CusF (b0573), outer membrane protein CusC (b0572), periplasmic adaptor protein CusB (b0574), and permease subunit CusA (b0575) in *Escherichia coli* (44), respectively. The coding products *I4W82_02515*, *I4W82_02520*, and *I4W82_04530* were homologous to those of the copper-responsive transcription factor CueR (b0487), copper transport ATPase CopA (b0484), and multicopper oxidase CueO (b0123) in the copper-resistant *Cue* system of *Escherichia coli* (44), respectively. A 27-bp CueR binding regulatory sequence was discovered in the −35−−10 promoter region of *copA* and *cueO* (45), indicating that, similar to that in *Escherichia coli*, both copA and cueO were regulated by CueR in the LDS17 cells. *I4W82_19230* and *I4W82_19235* probably code for the copper-resistant proteins CopC and CopD (46). In addition, we identified four genes (*I4W82_07005*, *I4W82_19070*, *I4W82_01330*, and *I4W82_04100*) belonging to the *Cut* system in the LDS17 genome, which have been reported to be associated with copper resistance in *Escherichia coli* (47). The encoded products of these four genes shared 82.1%, 70.6%, 83.4%, and 74.4% amino acid identity with CutA (b4137), CutC (b1874), CutE (b0657), and CutF (b0192) in *Escherichia coli*, respectively.

An arsenic resistance-related gene cluster, *arsRBC,* was identified in the LDS17 genome (Table S4; Fig. 8). The coding products of this gene cluster included ArsR (I4W82_13595), a regulatory factor that regulates the transcription of the *ars* operon; arsenite efflux transporter ArsB (*I4W82_13600*); and arsenate reductase ArsC (*I4W82_13605*), which catalyzes the conversion of arsenate to arsenite.

We identified two genes (*I4W82_02730* and *I4W82_10840*) associated with nickel/cobalt and zinc resistance, respectively, in the LDS17 genome (Table S4). The gene *I4W82_02730* encodes the nickel/cobalt efflux protein, RcnA, which is responsible for transporting extracellular nickel and cobalt. The gene *I4W82_10840* codes for the zinc-exporting P-type ATPase, ZntA (48). In addition, the Zn(II)-responsive transcriptional regulator gene *zntR* (*I4W82_11520*) was identified in the LDS17 genome.

### LDS17 confers resistance to the HMs copper, zinc, and nickel

**Fig 10:**
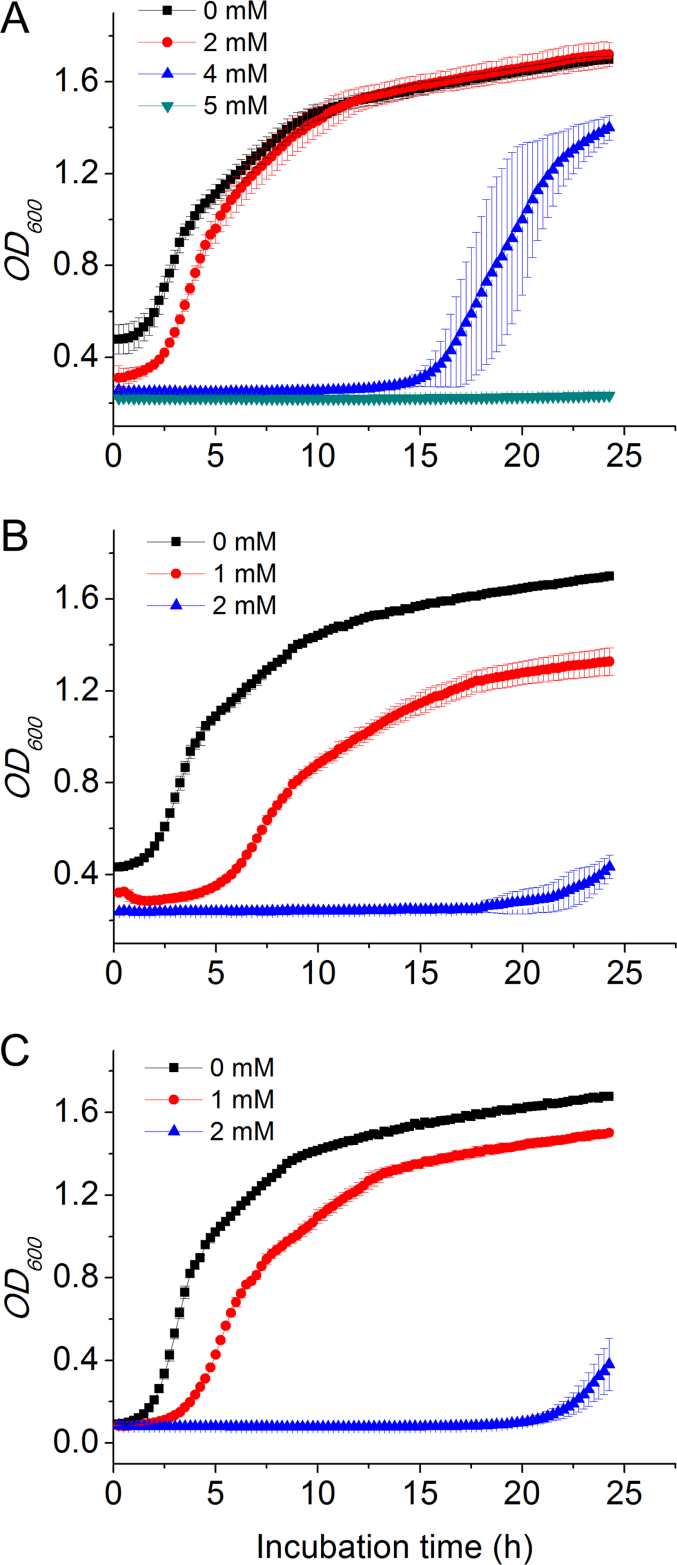
The growth curves of *Klebsiella michiganensis* LDS17 in LB media containing various concentrations of HM salts. (A) CuSO_4_; (B) ZnSO_4_; and (C) NiCl_2_.

Based on the identification of HM resistance genes through the genomics analysis, we measured the growth curves of the LDS17 strains in LB media supplemented with different concentrations of HM salts (CuSO_4_, ZnSO_4_, CoCl_2_, and NiCl_2_) to verify the tolerance of LDS17 to these HM salts. LDS17 showed no significant resistance to CoCl_2_. LDS17 exhibited varying degrees of resistance to CuSO_4_, ZnSO_4_, and NiCl_2_. The highest concentrations of HM salts for LDS17 growth were CuSO_4_: 4 mM, ZnSO_4_: 2 mM, and NiCl_2_: 1 mM, respectively (Fig. 10).

### Identification of LuxR solo, which putatively senses plant-derived signals

**Fig 11:**
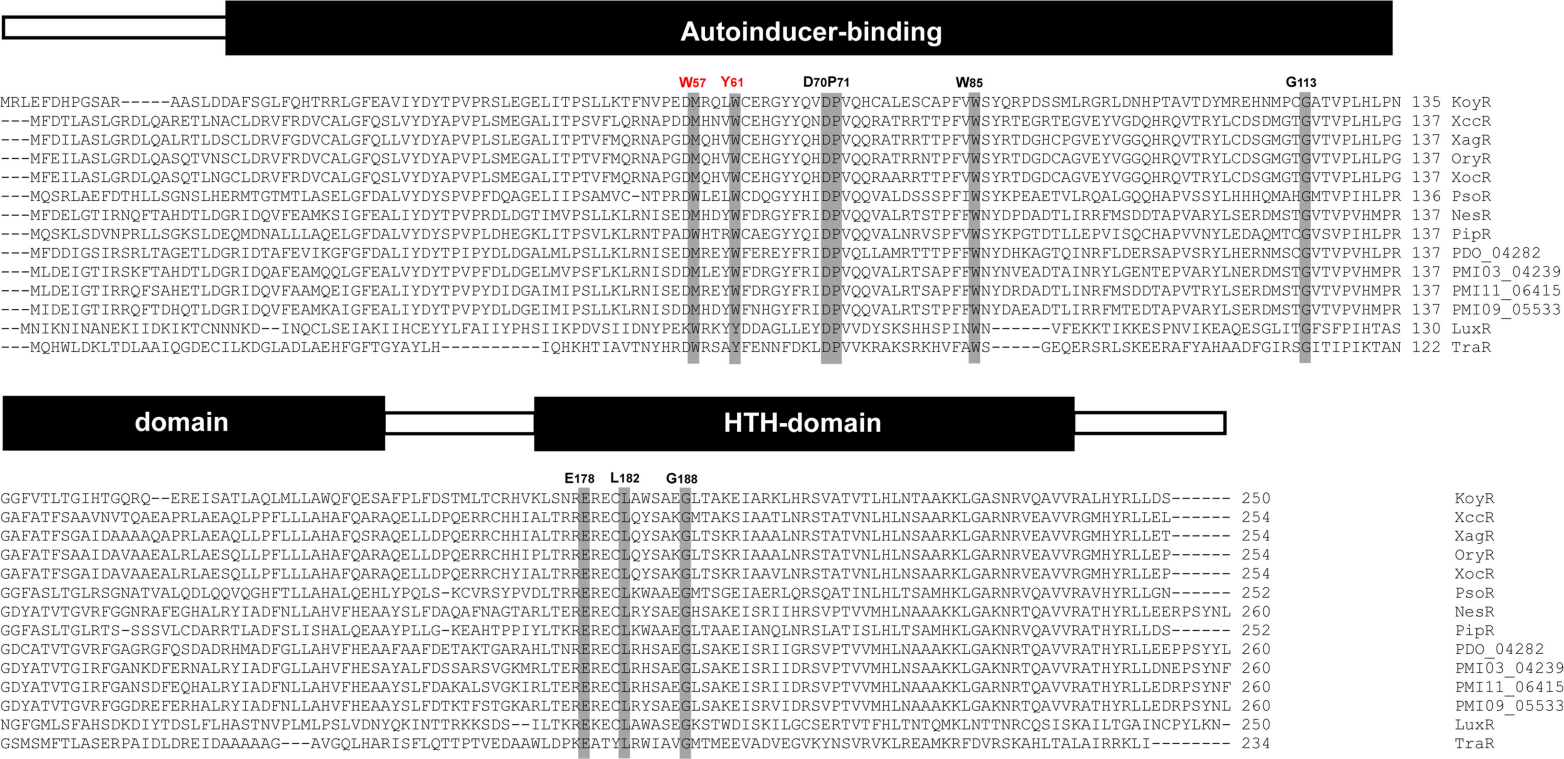
Amino acid sequence alignment of LuxR solo family proteins. The source of each LuxR and its corresponding gene accession number are as follows. XccR: *Xanthomonas campestris* pv. *campestris* 8004, XC_1295; XagR: *Xanthomonas axonopodis* pv. *glycines* 12-2, A9D66_16010; OryR: *Xanthomonas oryzae* pv. *oryzae* PXO86, AZ54_18140; XocR: *Xanthomonas oryzae* pv. *oryzicola* RS105, ACU12_06325; PsoR: *Pseudomonas fluorescens* Pf5, PFL_5298; NesR: *Sinorhizobium meliloti* 1021, SMc04032; PipR: *Pseudomonas* sp. GM79, PMI36_04623; PDO_04282: *Rhizobium* sp. PDO1-076, PDO_04282; PMI03_04239: *Rhizobium* sp. AP16, PMI03_04239; PMI11_06415: *Rhizobium* sp. CF142, PMI11_06415; PMI09_05533: *Rhizobium* sp. CF122, PMI09_05533. All of the above belong to LuxR solos in PAB. LuxR: *Aliivibrio fischeri* ES114, VF_A0925; TraR: *Agrobacterium tumefaciens* C58, Atu6134. Both LuxR and TraR are typical LuxR in bacterial QS systems.

**Fig 12:**
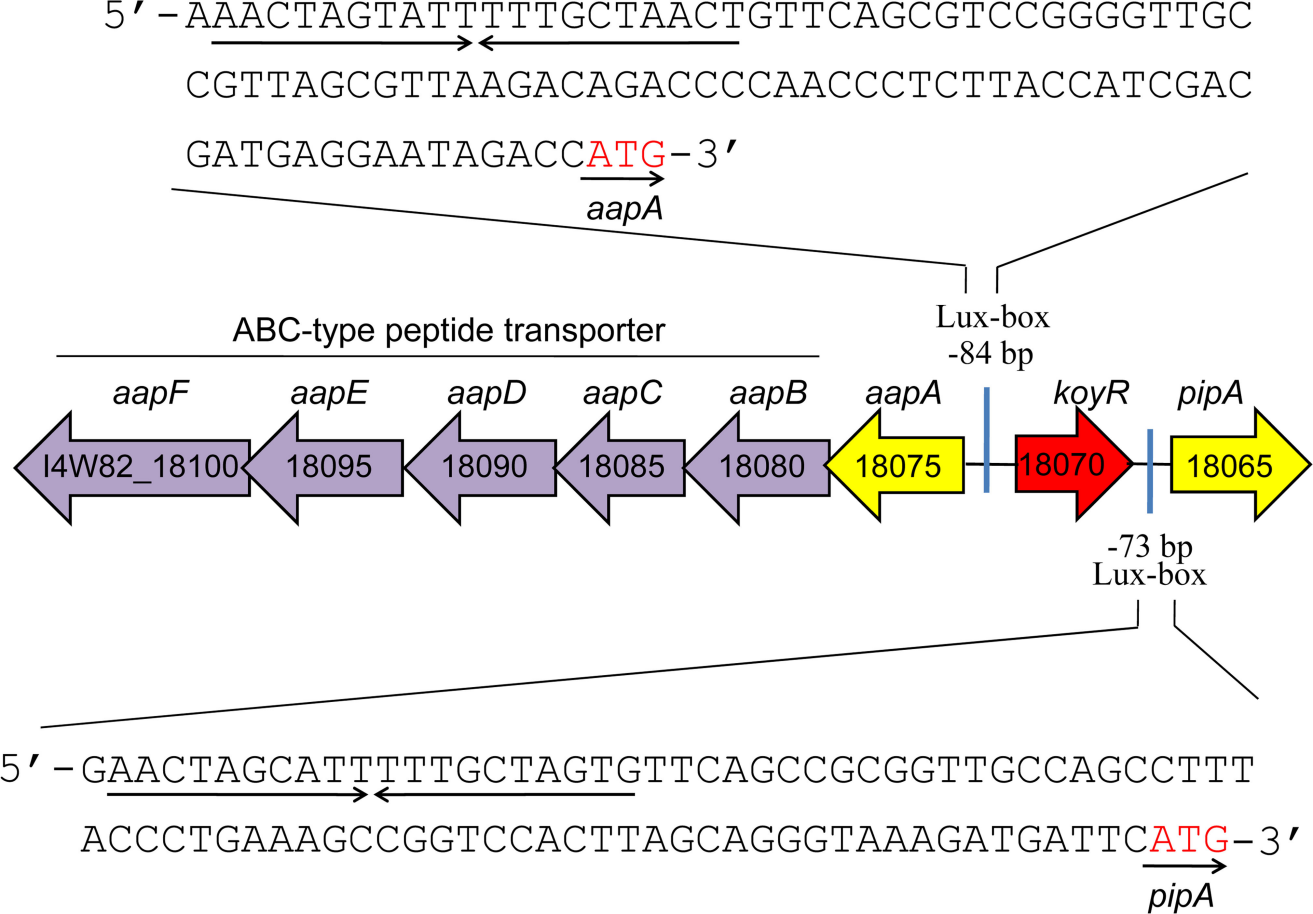
Gene arrangement on both sides of *koyR* in *Klebsiella michiganensis* LDS17.

We identified a gene (*I4W82_18070*) encoding a transcription factor called “LuxR solo” on the LDS17 genome, which is homologous to the LuxR family proteins found in bacterial acyl-homoserine lactone (AHL)-type quorum-sensing (QS) systems (49, 50). The *luxR solo* gene is not usually linked to the AHL synthetase *luxI* gene but exists independently (51). The *luxR solo* gene found in the LDS17 genome was named *koyR*. According to an amino acid sequence analysis, KoyR was predicted to belong to the LuxR solo subgroup, which senses plant-derived signals rather than AHL signals (49). The most typical representatives of the LuxR subgroup are OryR and XocR in *Xanthomonas oryzae* (52, 53), XccR in *Xanthomonas campestris* (54), XagR in *Xanthomonas axonopodis* (55), PipR in *Pseudomonas* sp. GM79 (56), *PsoR in Pseudomonas fluorescens* (57), and NesR in *Sinorhizobium meliloti* (58). KoyR is structurally similar to OryR, with M57 and W61 replacing W57 and Y61 [TraR in *Agrobacterium tumefaciens* as a reference (59)], which are highly conserved in AHL-responsive LuxR (Fig. 11). Both sides of the *koyR* gene contained an aproline iminopeptidase-encoding gene (*pip*). Both of these *pip* gene promoter regions contained inverted repeat DNA sequences that were similar to that of the lux-box. LuxR solo bound by plant-derived signals can recognize lux-box to activate *pip* gene transcription (Fig. 12). There is an ABC transporter-coding gene cluster upstream of the *koyR* gene (Fig. 12). The arrangement of *koyR* and its neighboring genes is highly similar to that of *pipR*, a plant-responsive *luxR* solo in the cottonwood tree endophyte *Pseudomonas* sp. GM79 (56). KoyR was clustered in a relatively separate branch with the currently reported plant-responsive LuxR solos of plant-associated bacteria (PAB) in the phylogenetic tree constructed based on the amino acid sequences of the LuxR family proteins (Fig. S4). Therefore, we believe that KoyR is a member of the plant-responsive LuxR solos family.

## DISCUSSION

In this study, we isolated the PGPB LDS17 strain with high ACC deaminase activity from the *Codonopsis pilosula* rhizosphere. LDS17 inoculation not only promoted the growth of *Codonopsis pilosula* seedlings but also improved soil quality by increasing rhizosphere soil enzyme activity and functional diversity of microbial communities, thereby providing a good soil environment for *Codonopsis pilosula* growth. Furthermore, the whole-genome analysis of LDS17 revealed many genes associated with plant growth promotion and HM resistance. LDS17 was identified as a strain belonging to *Klebsiella michiganensis*. Although many PGPB belonging to the *Klebsiella* genus have been identified (60–62), there have been few reports on *Klebsiella michiganensis* PGPB (63). In addition, while the GenBank database contains over 500 genomic records for *Klebsiella michiganensis*, the vast majority of which are pathogenic bacteria, only six strains describe PGP characteristics (*Klebsiella michiganensis* strain SA2, FR 1, FR 3, FR 19, FR 89, and FR 129), and there is no analysis of plant growth related genes in these six genomes. As a result, the whole-genome sequence of *Klebsiella michiganensis* LDS17 enriched the few current genome databases of *Klebsiella michiganensis* with PGP abilities. An in-depth analysis of its PGP and HM-resistant genes provided an important theoretical basis for future research into the molecular mechanisms underlying the plant growth promotion and HM resistance yielded by the strain.

The colonization of the rhizosphere and/or plant tissue by PGPB is crucial for plant growth (64, 65). PGPB with good rhizosphere and plant tissue colonization performance are typically expected to be highly efficient in allowing for plant growth (19). Rhizosphere and root colonization experiments showed that LDS17 could stably colonize the rhizosphere and root tissues of *Codonopsis pilosula* plants within 40 days of inoculation (Fig. S5). These results suggest that LDS17 is a good colonizer, is compatible with *Codonopsis pilosula* plants, and exhibits PGP function.

Our study revealed that LDS17 inoculation increased invertase and urease activity, the number of culturable microorganisms, and the functional diversity of microbial communities in *Codonopsis pilosula* rhizosphere soil. Soil is a substrate for plant growth, and its quality has a direct impact on plant growth and development. Soil enzymatic activity, microbial communities, and functional diversity are important indicators of soil quality (66, 67). Kong and Liu (19) summarized the effects of PGPB inoculation on the ecology of plant rhizosphere microorganisms and soil function and speculated that PGPB may indirectly promote plant growth by changing the composition and function of rhizosphere microbial communities. Therefore, we hypothesized that LDS17 inoculation provides a favorable rhizosphere ecological environment for the growth of *Codonopsis pilosula* plants by improving the rhizosphere soil quality to some extent.

ACC deaminase-producing bacteria have been reported to improve plant resistance to abiotic stressors (such as drought stress) (10). Therefore, we measured the malondialdehyde (MDA) content and antioxidant enzyme (SOD, POD, and CAT) activity in the leaves of *Codonopsis pilosula* under drought stress conditions (the methods were described in the supplemental material). Inoculation with strain LDS17 resulted in a significant decrease in the MDA content (up to 17.4%) compared to that in the uninoculated control group. Additionally, the SOD, POD, and CAT activities of the LDS17 inoculation treatment group increased by 6.25%, 10.87%, and 14.69%, respectively, compared to those in the control group (Table S5). This indicates that LDS17 can help *Codonopsis pilosula* improve its resistance to drought stress by lowering MDA levels in the leaf tissue and increasing antioxidant enzyme activity.

Todorovic and Glick (68) reported that Glu295 and Leu322 mutations in five key amino acid residues of the AcdS sequence resulted in the loss of ACC deaminase activity. However, these two critical amino acid residues were missing from the predicted ACC deaminase sequence (*I4W82_18900*) of LDS17. We previously reported a *Pseudomonas putida* strain, LWPZF, with the ability to produce ACC deaminase (26). Liu et al. (69) further isolated a *Serratia marcescens* strain, JW-CZ2, with high ACC deaminase activity. The amino acid sequence of ACC deaminase predicted in the genomes of these two strains also lacked Glu295 and Leu322 but contained the Lys51, Ser78, and Tyr294 amino acid residues. Therefore, it remains to be determined whether Glu295 and Leu322 are required for ACC deaminase activity in different strains.

The plant growth-promotion characterization assay showed that LDS17 dissolved inorganic P (Fig. 2A). In the LDS17 genome, we identified a gene encoding glucose dehydrogenase, which catalyzes the conversion of glucose to gluconic acid. Normally, the cofactor PQQ is required for glucose dehydrogenase function (37). However, the PQQ synthesis gene cluster was not detected in the LDS17 genome. Further research is needed to (i) determine whether glucose dehydrogenase can catalyze glucose to produce gluconic acid independently of PQQ and thus play a role in the dissolution of P by LDS17 and (ii) determine whether there are other P-dissolving mechanisms involving LDS17.

Excessive HM residues in herbal plants can directly cause health risks and are a common concern (70). Excessive HMs in herbal plants are primarily the result of excess HMs remaining in the soils in which the plants grow. Many PGPB have been reported to help plants improve HM resistance through their own HM resistance mechanisms. These HM-resistant PGPB inoculants not only increase the biomasses of plants growing in HM-polluted environments but also significantly reduce the HM uptake by plant tissues, including roots and shoots (71–73). According to our genomic sequence analysis, the LDS17 strain carries multiple genes associated with HM resistance, including copper, nickel/cobalt, and zinc resistance. The experiments also confirmed that LDS17 is resistant to copper, zinc, and nickel. Therefore, LDS17 may be useful for the development of microbial fertilizers for herbal plants, including *Codonopsis pilosula*, under HM stress.

The known molecular mechanisms underlying copper resistance in Gram-negative bacteria mainly include the *Cue*, *Cus*, *Pco*, and *Cop* systems (44). We identified intact *Cue* and *Cus* systems, as well as a part of the *Cop* system, in the LDS17 genome. The complete *Cop* system consists of *copS*, *copR*, *copA*, *copB*, *copC*, and *copD* (44). We only found the *copC* and *copD* genes in the LDS17 genome. Based on the functional prediction of copper resistance-related genes in the LDS17 genome, we hypothesized that the copper resistance mechanism in LDS17 is as follows: CueR senses excess intracellular copper ions and activates the transcription of the ATPase gene *copA*. CopA transports excess intracellular copper ions into the periplasmic space. CopC binds excess univalent copper ions in the periplasmic space and transfers them to CueO, which oxidizes univalent copper ions to bivalent copper ions with low toxicity. Another portion of the monovalent copper ions is transported from the periplasmic space to the extracellular space by an efflux pump composed of CusA, CusB, and CusC in the *Cus* system, with the assistance of the periplasmic chaperone CusF (Fig. 9). Another system known as *Cut* has been reported to play an important role in regulating copper concentrations in *Escherichia coli* cells (47). The system includes six genes: *cutA*, *cutB*, *cutC*, *cutD*, *cutE*, and *cutF. CutC* and *cutF* have been identified as being involved in copper resistance (74). The functions of the remaining four genes remain unknown. We identified *cutA*, *cutC*, *cutE*, and *cutF* in the LDS17 genome. The precise roles of these four genes, particularly *cutA* and *cutE*, in the copper resistance process of LDS17 require further investigation.

RcnA reportedly exerts efflux effects on both nickel and cobalt (75). The HM resistance test revealed that LDS17 was only resistant to nickel and not to cobalt (Fig. 10). MdrH, an RcnA homolog found in *Pseudomonas putida*, also lacked resistance to cobalt (76). We hypothesized that the RcnA protein in LDS17 may contribute only to nickel resistance in combination with HM resistance experiments (Fig. 10).

Existing researches indicate that plant-responsive LuxR solos play an important role in the interactions between PAB and plants. LuxR solos are involved in the pathogenicity of *Xanthomonas* (49). There have been two reports on plant-responsive LuxR solos in plant-beneficial bacteria: PsoR in the biocontrol bacterium *Pseudomonas fluorescens* and NesR in *Sinorhizobium meliloti*. PsoR is related to the prevention of wheat root rot and damping-off caused by *Pythium ultimum* in *Pseudomonas fluorescens* (57). The absence of NesR in *Sinorhizobium meliloti* results in a significant reduction in the competitive nodulation ability of the species (58). These studies indicate that plant-responsive LuxR solos are important for biocontrol and plant growth promotion by plant-beneficial bacteria. Therefore, we hypothesize that KoyR is involved in the PGP effects of LDS17 on *Codonopsis pilosula* seedlings. The functions of KoyR are worth investigating in the future.

At present, lots of PGPB have been isolated from various sources; however, understanding their PGP mechanisms is critical for further utilization of these strains in microbial fertilizers. We examined the genes associated with plant growth promotion and HM resistance in the whole genome of LDS17. This not only laid the groundwork for further research into understanding the mechanisms by which LDS17 promotes plant growth and resistance to HM but may also serve as a point of reference for studies on PGPB belonging to *Klebsiella michiganensis*. The effects of LDS17 on the growth of *Codonopsis pilosula* seedlings and the improvement of rhizosphere soil quality should be due to the combined effects of various PGP characteristics. The molecular mechanisms regulating these PGP properties and their contribution to the growth-promoting process of *Codonopsis pilosula* need to be investigated further. The question remains as to whether KoyR plays a role in the interactions between LDS17 and *Codonopsis pilosula* plants, similar to other reported plant-responsive LuxR solos. How do HM resistance and other PGP characteristics work together to complete PGP tasks when plants are exposed to HM stress? The findings of this study provide an important foundation for future studies investigating these issues.

## Data Availability

The whole-genome sequence and 16S rRNA gene sequence of strain LDS17 have been submitted to GenBank under the accession numbers CP065338 and OR690259, respectively.

## SUPPLEMENTAL MATERIAL

Supplemental material spectrum.04056-23-s0001.docx Supplemental methods, Tables S1 to S5, and Fig. S1 to S5.

ASM does not own the copyrights to Supplemental Material that may be linked to, or accessed through, an article. The authors have granted ASM a non-exclusive, world-wide license to publish the Supplemental Material files. Please contact the corresponding author directly for reuse.
