# Supplementary material for: Effects of Klebsiella michiganensis LDS17 on Codonopsis pilosula growth, rhizosphere soil enzyme activities, and microflora, and genome-wide analysis of plant growth-promoting genes
Source: Microbiol Spectr. 2024 Apr 2;12(5):e04056-23. doi: 10.1128/spectrum.04056-23 (PMC11064500; doi:10.1128/spectrum.04056-23)
Supplement: Supplemental material — Supplemental methods, Tables S1 to S5, and Fig. S1 to S5. [file spectrum.04056-23-s0001.docx]

**Effects of *Klebsiella michiganensis* LDS17 on *Codonopsis pilosula* growth, rhizosphere soil enzyme activities and microflora, and genome-wide analysis of plant growth-promoting genes**

Tingting Jin^#^, Jiahong Ren^#^*, Bianxia Bai*, Wei Wu, Yongqing Cao, Jing Meng, Lihui Zhang

Department of Life Sciences, Changzhi University, Changzhi 046011, PR China

^#^Tingting Jin and Jiahong Ren contributed equally to this article

*Corresponding author: Jiahong Ren, renjiahong76@hotmail.com, Bianxia Bai, baibianxia08@126.com

**Methods**

**Antagonistic tests against pathogenic fungi**

The antifungal activity of LDS17 against the phytopathogenic fungi (*Rhizoctonia solani*, *Colletotrichum camelliae*, *Cytospora chrysosperma*, *Phomopsis macrospore*, *Colletotrichum gloeosporioides*, *Fusicoccum aesculi*, *Rhizoctonia* sp., *Botryosphaeria dothidea*) was evaluated using Petri dishes (9 cm in diameter). Briefly, a 5 mm diameter disk of pathogenic fungi was inoculated in the center of potato dextrose agar (PDA) plates. Colonies of LDS17 were streaked 1 cm from the margin of the PDA plates. Control plates, only incubated with each pathogenic fungus, were prepared. The plates were kept at 28 °C for incubation. When the fungal hyphae on the control plate reach the Petri dishes' edge, the percent inhibition (PI%) was calculated: PI% = [(A-B)/A] × 100. A and B are mycelial diameters on the control and experimental plates, respectively.

**Colonization assay**

The pGFP4412 plasmid (neomycin and ampicillin resistance, denoted by Professor Sanfeng Chen from China Agricultural University) containing the green fluorescent protein (GFP) gene was transformed into LDS17 cells to obtain a GFP-labeled LDS17 strain. The LDS17/pGFP4412 inoculant was then inoculated into the rhizosphere of 2-month-old *Codonopsis pilosula* seedlings planted in sterilized soil. The growth conditions for the *Codonopsis pilosula* seedlings were the same as those used in the pot culture experiments. Thirty replicates were used for each treatment group. The rhizosphere soil and roots of the seedlings were collected on days 5, 10, 15, 20, 25, 30, 35, and 40 after inoculation for LDS17/pGFP4412 cell detection. The roots were surface-sterilized with 75% ethanol for 3 min, followed by six rinses with sterile water, and thoroughly homogenized in a mortar. The gradient dilutions (with sterile water) of the rhizosphere soils and homogenized roots were spread on LB agar plates supplemented with neomycin (7 µg mL^–1^) and ampicillin (100 µg mL^–1^), respectively, and incubated for 2–3 days. The colonization of LDS17/pGFP4412 in the soil or roots was measured as CFU g^–1^ of soil or CFU g^–1^ of root.

After 40 days of inoculation, the roots of the *Codonopsis pilosula* seedlings were washed with sterile water, cut lengthwise into 1-cm segments, and placed on a slide. The colonization of the GFP-labeled LDS17 strain on the root surface and inside was observed using fluorescence microscopy (Leica, DMC6200).

**Drought stress treatment**

The LDS17 bacterial suspensions were inoculated into the rhizosphere of two-month-old *Codonopsis pilosula* seedlings planted in sterilized soil. Seedlings treated with equal volumes of distilled water under the same conditions were used as control. Then seedlings were cultured under the following conditions: (1) normal water treatment, soil moisture content was maintained at 70% of the maximum water holding capacity, and (2) drought stress treatment, soil moisture content was maintained at 35% of the maximum water holding capacity. Each treatment was replicated thirty times. All seedlings were grown in greenhouse at 25 °C (14 hours light, 10 hour dark time). Malondialdehyde (MDA) content and the activities of SOD, CAT, and POD in each seedling were evaluated after 20 days of treatment.

**MDA content and antioxidant enzyme activity measurement**

Fresh leaves (0.1 g) were frozen at −80 °C and ground into a homogenate in 5 mL 0.05 mol/L sodium phosphate buffer (pH7.0) and centrifuged at 10,000 × *g* for 20 min at 4 °C. The supernatant was stored at 4 °C as enzyme extracts for further analysis. MDA content and antioxidant enzyme activity were determined according to the description of Fu and Huang (2001).

**References**

Fu J, Huang B. 2001. Involvement of antioxidants and lipid peroxidation in the adaptation of two cool-season grasses to localized drought stress. Environ Exp Bot 45:105-114.

**TABLE S1 The detailed information of the sampling sites**

| **Sample number** | **Sampling sites** | **Elevation (m)** | **Coordinates** |
| --- | --- | --- | --- |
| 1 | Fotangling Village, Pingshun County, Changzhi City | 1170.01 | 36° 10′ 25.2″ N  113° 48′ 22.5″ E |
| 2 | Dongpo Village, Zezhou County, Jincheng City | 1214.22 | 35° 39′ 90.8″ N  112° 93′ 39.9″ E |
| 3 | Xizhashui Village, Lingchuan County, Jincheng City | 1186.30 | 35° 41′ 14.7″ N  113° 25′ 55.3″ E |
| 4 | Renjiazhuang Village, Shangdang District, Changzhi City | 1055.22 | 36° 24′ 68″ N  113° 86′ 19.4″ E |

**TABLE S2 Comparisons between the ANI of *Klebsiella michiganensis* LDS17 and other closely related strains**

| **Strains** | **Genome accesion** | **ANI** |
| --- | --- | --- |
| *Klebsiella michiganensis* W14 (T) | GCA_002925905.1 | 99.07% |
| *Klebsiella oxytoca* FDAARGOS_500 | GCA_003812925.1 | 91.92% |
| *Klebsiella grimontii* 06D021 (T) | GCA_900200035.1 | 93.36% |
| *Klebsiella pasteurii* Sb-24 | GCA_018139045.1 | 93.49% |
| *Enterobacter huaxiensis* 090008 (T) | GCA_003594935.2 | 81.55% |
| *Yokenella regensburgei* ATCC 49455 (T) | GCA_000735455.1 | 81.03% |
| *Lelliottia jeotgali* PFL01 (T) | CP018628 | 80.97% |
| *Klebsiella spallanzanii* SB6411 | GCA_902158555.1 | 88.44% |
| *Enterobacter bugandensis* EB-247 (T) | NZ_LT992502 | 81.52% |
| *Enterobacter cancerogenus* JY65 | NZ_CP081105 | 81.28% |
| *Phytobacter ursingii* ATCC 27989 (T) | GCA_901456055.1 | 80.58% |
| *Pseudescherichia vulneris* NBRC 102420 (T) | GCA_000759795.1 | 80.74% |
| *Kosakonia oryzendophytica* REICA_082 (T) | GCA_900094925.1 | 80.16% |
| *Raoultella terrigena* JH01 | CP050508 | 84.20% |
| *Citrobacter gillenii* MBT-C3 | GCA_003429605.1 | 80.89% |
| *Klebsiella quasipneumoniae* subsp. *Similipneumoniae* 07A044 (T) | GCA_000613225.1 | 83.87% |
| *Citrobacter pasteurii* CIP 55.13 (T) | GCF_000826205.1 | 80.94% |
| *Klebsiella aerogenes* KCTC 2190 (T) | CP002824 | 83.66% |
| *Enterobacter chuandaensis* 090028 (T) | GCA_003594915.1 | 81.39% |
| *Raoultella ornithinolytica* NCTC9164 | GCA_901421005.1 | 84.15% |
| *Klebsiella huaxiensis* WCHKl090001 (T) | GCA_003261575.2 | 87.36% |
| *Citrobacter freundii* FDAARGOS_549 | GCA_003812345.1 | 81.13% |
| *Klebsiella pneumoniae* subsp. *Rhinoscleromatis* ATCC 13884 (T) | GCA_000163455.1 | 83.69% |

**TABLE S3 Accession numbers of housekeeping genes used for phylogenetic tree construction**

| **Strains** | **Genome accesion** | ***recA*** | ***gyrB*** | ***rpoA*** | ***rpoB*** | ***rpoC*** | ***rpoD*** |
| --- | --- | --- | --- | --- | --- | --- | --- |
| LDS17 | CP065338 | I4W82_15160 | I4W82_09320 | I4W82_11505 | I4W82_07830 | I4W82_07825 | I4W82_12630 |
| *Klebsiella michiganensis* THO-011 | GCA_015139575.1 | THOKLE011_11830 | THOKLE011_00040 | THOKLE011_04570 | THOKLE011_51250 | THOKLE011_51240 | THOKLE011_06700 |
| *Klebsiella oxytoca* FDAARGOS_500 | GCA_003812925.1 | EGY21_10565 | EGY21_16905 | EGY21_14775 | EGY21_18415 | EGY21_18420 | EGY21_13365 |
| *Klebsiella grimontii* 06D021 (T) | GCA_900200035.1 | KOSB73_20107 | KOSB73_110044 | KOSB73_60016 | KOSB73_160012 | KOSB73_160013 | KOSB73_40228 |
| *Klebsiella pasteurii* Sb-24 | GCA_018139045.1 | KCG39_12535 | KCG39_18425 | KCG39_16110 | KCG39_19880 | KCG39_19885 | KCG39_14950 |
| *Enterobacter huaxiensis* 090008 (T) | GCA_003594935.2 | D5067_0005250 | D5067_0000020 | D5067_0002135 | D5067_0022100 | D5067_0022095 | D5067_0003275 |
| *Yokenella regensburgei* ATCC 49455 (T) | GCA_000735455.1 | GYRE_02186 | GYRE_01006 | GYRE_01371 | GYRE_00623 | GYRE_00622 | GYRE_02655 |
| *Lelliottia jeotgali* PFL01 (T) | CP018628 | LJPFL01_3216 | LJPFL01_0004 | LJPFL01_3832 | LJPFL01_0197 | LJPFL01_0198 | LJPFL01_3571 |
| *Klebsiella spallanzanii* SB6411 | GCA_902158555.1 | SB6411_03851 | SB6411_00868 | SB6411_05770 | SB6411_05738 | SB6411_05737 | SB6411_02588 |
| *Enterobacter bugandensis* EB-247 (T) | NZ_LT992502 | DG357_RS17835 | DG357_RS00020 | DG357_RS20700 | DG357_RS01115 | DG357_RS01120 | DG357_RS19560 |
| *Enterobacter cancerogenus* JY65 | NZ_CP081105 | K3T75_RS17925 | K3T75_RS01190 | K3T75_RS21160 | K3T75_RS00100 | K3T75_RS00095 | K3T75_RS19690 |
| *Phytobacter ursingii* ATCC 27989 (T) | GCA_901456055.1 | PUATCC27989T_00118 | PUATCC27989T_04425 | PUATCC27989T_04966 | PUATCC27989T_04107 | PUATCC27989T_04106 | PUATCC27989T_05237 |
| *Pseudescherichia vulneris* NBRC 102420 (T) | GCA_000759795.1 | EV102420_02_00540 | EV102420_20_00320 | EV102420_40_00160 | EV102420_35_00210 | EV102420_35_00200 | EV102420_08_01880 |
| *Kosakonia oryzendophytica* REICA_082 (T) | GCA_900094925.1 | GA0061071_11224 | GA0061071_11636 | GA0061071_12331 | GA0061071_12110 | GA0061071_12111 | GA0061071_11519 |
| *Raoultella terrigena* JH01 | CP050508 | HCK03_05620 | HCK03_00020 | HCK03_02055 | HCK03_25000 | HCK03_24995 | HCK03_03340 |
| *Citrobacter gillenii* MBT-C3 | GCA_003429605.1 | DZA29_17055 | DZA29_10380 | DZA29_25290 | DZA29_25000 | DZA29_24995 | DZA29_11550 |
| *Klebsiella quasipneumoniae* subsp. *Similipneumoniae* 07A044 (T) | GCA_000613225.1 | SB30_280111 | SB30_370039 | SB30_330016 | SB30_30011 | SB30_30012 | SB30_320065 |
| *Citrobacter pasteurii* CIP 55.13 (T) | GCF_000826205.1 | CIT5513_RS13415 | CIT5513_RS08515 | CIT5513_RS11870 | CIT5513_RS05570 | CIT5513_RS05575 | CIT5513_RS04215 |
| *Klebsiella aerogenes* KCTC 2190 (T) | CP002824 | EAE_01520 | EAE_07020 | EAE_04830 | EAE_08125 | EAE_08130 | EAE_03690 |
| *Enterobacter chuandaensis* 090028 (T) | GCA_003594915.1 | D5066_13015 | D5066_17835 | D5066_22475 | D5066_22230 | D5066_22235 | D5066_15030 |
| *Raoultella ornithinolytica* NCTC9164 | GCA_901421005.1 | NCTC9164_01306 | NCTC9164_00203 | NCTC9164_00632 | NCTC9164_05053 | NCTC9164_05052 | NCTC9164_00843 |
| *Klebsiella huaxiensis* WCHKl090001 (T) | GCA_003261575.2 | DA718_06485 | DA718_00020 | DA718_02640 | DA718_27875 | DA718_27870 | DA718_03920 |
| *Citrobacter freundii* FDAARGOS_549 | GCA_003812345.1 | EGX89_08700 | EGX89_13775 | EGX89_11705 | EGX89_15150 | EGX89_15155 | EGX89_10525 |
| *Klebsiella pneumoniae* subsp. *Rhinoscleromatis* ATCC 13884 (T) | GCA_000163455.1 | HMPREF0484_4048 | HMPREF0484_0722 | HMPREF0484_5201 | HMPREF0484_3901 | HMPREF0484_3900 | HMPREF0484_2446 |

**TABLE S4 Genes involved in plant growth promotion, and heavy metal resistance in the *Klebsiella michiganensis* LDS17 genome**

| **LDS17 ORF ID (I4W82_)** | **Gene** | **Function** |
| --- | --- | --- |
| **IAA biosynthesis** |  |  |
| 16760 |  | Indolepyruvate decarboxylase (EC 4.1.1.74) |
| 22600 |  | Amidase (EC 3.5.1.4) |
| 22605 | *nthA* | Nitrile hydratase subunit alpha (EC 4.2.1.84) |
| 22610 | *nthB* | Nitrile hydratase subunit beta (EC 4.2.1.84) |
| **ACC deaminase activity** |  |  |
| 18900 |  | Putative 1-aminocyclopropane-1-carboxylate deaminase |
| **P solubilization** |  |  |
| 04525 |  | PQQ-dependent dehydrogenase (EC 1.1.5.2) |
| **Enterobactin synthesis** |  |  |
| 01675 | *entH* | Proofreading thioesterase EntH |
| 01680 | *entA* | 2, 3-dihydro-2, 3-dihydroxybenzoate dehydrogenase EntA |
| 01685 | *entB* | Enterobactin biosynthesis bifunctional isochorismatase/aryl carrier protein EntB |
| 01690 | *entE* | (2,3-dihydroxybenzoyl) adenylate synthase EntE |
| 01695 | *entC* | Isochorismate synthase EntC |
| 01700 | *fepB* | Fe^2+^-enterobactin ABC transporter substrate-binding protein |
| 01725 | *entS* | Enterobactin transporter EntS |
| 01730 | *fepD* | Fe^3+^-siderophore ABC transporter permease |
| 01735 | *fepG* | Iron-enterobactin ABC transporter permease |
| 01740 | *fepC* | Iron-enterobactin ABC transporter ATP-binding protein |
| 01745 | *entF* | Enterobactin non-ribosomal peptide synthetase EntF |
| 01750 | *ybdZ* | MbtH family NRPS accessory protein |
| 01755 | *fes* | Enterochelin esterase |
| 01760 | *fepA* | TonB-dependent siderophore receptor |
| 01765 | *entD* | Enterobactin synthase subunit EntD |
| **Nitrogen fixation** |  |  |
| 18370 | *nifQ* | Nitrogen fixation protein NifQ |
| 18375 | *nifB* | Nitrogenase cofactor biosynthesis protein NifB |
| 18380 | *nifA* | Nif-specific transcriptional activator NifA |
| 18385 | *nifL* | Nitrogen fixation negative regulator NifL |
| 18390 | *nifF* | Flavodoxin |
| 18395 | *nifM* | Nitrogen fixation protein NifM |
| 18400 | *nifZ* | Nitrogen fixation protein NifZ |
| 18405 | *nifW* | Nitrogenase-stabilizing/protective protein NifW |
| 18410 | *nifV* | Homocitrate synthase |
| 18415 | *nifS* | Cysteine desulfurase NifS |
| 18420 | *nifU* | Fe-S cluster assembly protein NifU |
| 18425 | *nifX* | Nitrogen fixation protein NifX |
| 18430 | *nifN* | Nitrogenase iron-molybdenum cofactor biosynthesis protein NifN |
| 18435 | *nifE* | Nitrogenase iron-molybdenum cofactor biosynthesis protein NifE |
| 18440 | *nifY* | Nitrogen fixation protein NifY |
| 18445 | *nifT* | Putative nitrogen fixation protein NifT |
| 18450 | *nifK* | Nitrogenase molybdenum-iron protein subunit beta |
| 18455 | *nifD* | Nitrogenase molybdenum-iron protein alpha chain |
| 18460 | *nifH* | Nitrogenase iron protein |
| 18465 | *nifJ* | Pyruvate:ferredoxin (flavodoxin) oxidoreductase |
| **Heavy metal resistance** | |  |
| 02515 | *cueR* | Cu(I)-responsive transcriptional regulator |
| 02520 | *copA* | Copper-exporting P-type ATPase CopA |
| 04530 | *cueO* | Multicopper oxidase CueO |
| 07005 | *cutA* | Divalent cation tolerance protein CutA |
| 19070 | *cutC* | Copper homeostasis protein CutC |
| 01330 | *cutE* | Apolipoprotein N-acyltransferase |
| 04100 | *cutF* | Envelope stress response activation lipoprotein NlpE |
| 19230 |  | CopC domain-containing protein |
| 19235 | *copD* | Copper homeostasis membrane protein CopD |
| 21820 | *cusF* | Copper-binding protein |
| 21825 | *cusC* | TolC family protein |
| 21830 | *cusB* | Efflux RND transporter periplasmic adaptor subunit |
| 21835 | *cusA* | Efflux RND transporter permease subunit |
| 13595 | *arsR* | ArsR family transcriptional regulator |
| 13600 | *arsB* | Arsenite efflux transporter membrane subunit ArsB |
| 13605 | *arsC* | Glutaredoxin-dependent arsenate reductase |
| 02730 | *rcnA* | Nickel/cobalt efflux protein RcnA |
| 10840 | *zntA* | Zn(II)-exporting P-type ATPase |
| 11520 | *zntR* | Zn(II)-responsive transcriptional regulator |

**TABLE S5 *Klebsiella michiganensis* LDS17 induced oxidative stress response in** *Codonopsis pilosula* **seedlings under drought stress**

| **Treatments** | **MDA (µmol/g FW)** | **SOD (U/g FW/h)** | **POD (U/g FW/min)** | **CAT (U/g FW/min)** |
| --- | --- | --- | --- | --- |
| Control | 3.33±0.14^b^ | 798.00±18.03^c^ | 253.33±7.04^c^ | 10.96±0.13^b^ |
| Control+LDS17 | 3.26±0.16^b^ | 820.00±12.69^b^ | 261.33±6.81^b^ | 10.98±0.09^b^ |
| Drought water | 3.62±0.10^a^ | 800.67±10.11^c^ | 260.67±4.44^b^ | 11.37±0.09^b^ |
| Drought water+LDS17 | 2.99±0.01^c^ | 850.67±7.28^a^ | 289.00±8.19^a^ | 13.03±0.76^a^ |

Values are means ± SD, different letters indicate significant differences (*P* < 0.05).


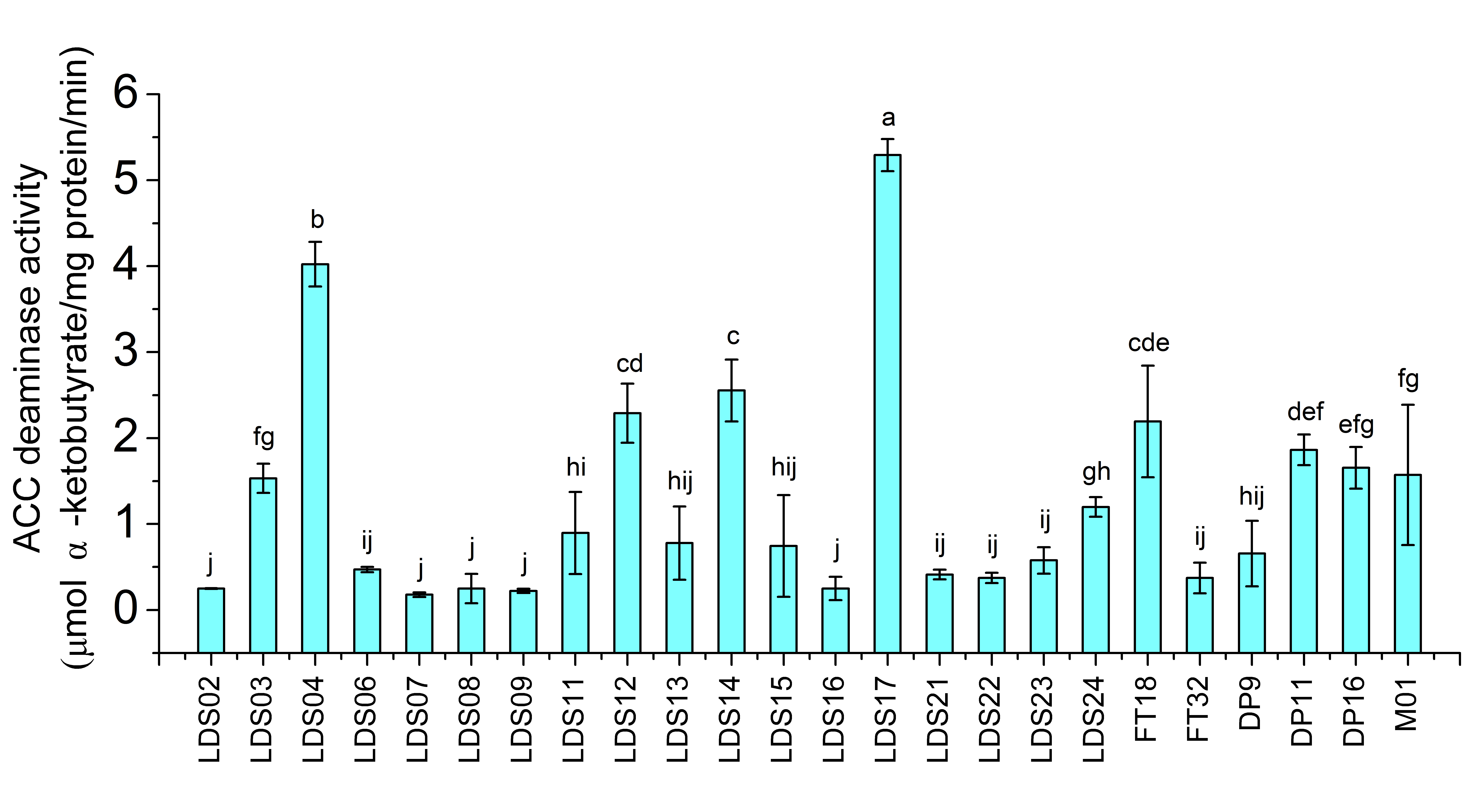


**FIG S1 ACC deaminase activities of 24 isolates from the *Codonopsis pilosula* rhizosphere.** Different letters in the column indicate significant differences (*P* < 0.05).

*Klebsiella oxytoca* JCM 1665^T^ (AB004754)

*Klebsiella pasteurii* SPARK 836 C1^T^ (MN091366)

*Klebsiella grimontii* 06D021^T^ (FZTC01000044)

*Klebsiella spallanzanii* SPARK 775 C1^T^ (MN091365)

**LDS17** (OR690259)

*Klebsiella michiganensis* W14^T^ (JQ070300)

*Enterobacter huaxiensis* 090008^T^ (MK049964)

*Kosakonia oryzendophytica* REICA 082^T^ (JF795011)

*Enterobacter chuandaensis* 090028^T^ (MK049966)

*Pseudescherichia vulneris* NBRC 102420^T^ (BBMZ01000044)

*Enterobacter bugandensis* EB-247^T^ (FYBI01000003)

*Enterobacter cancerogenus* ATCC 33241^T^ (FYBA01000020)

*Yokenella regensburgei* ATCC 49455^T^ (JMPS01000045)

*Klebsiella huaxiensis* WCHKl090001^T^ (MH179329)

*Phytobacter ursingii* ATCC 27989^T^ (FJ611881)

*Citrobacter bitternis* SKKUI-TP7^T^ (KJ817168)

*Lelliottia jeotgali* PFL01^T^ (KX709881)

*Citrobacter freundii* DSM 30039^T^ (AJ233408)

*Citrobacter gillenii* CDC 4693-86^T^ (AF025367)

*Citrobacter pasteurii* CIP 55.13^T^ (CDHL01000036)

*Klebsiella quasipneumoniae* subsp. *similipneumoniae* 07A044^T^ (CBZR010000040)

*Klebsiella pneumoniae* subsp. *rhinoscleromatis* ATCC 13884^T^ (ACZD01000038)

*Raoultella terrigena* ATCC 33257^T^ (Y17658)

*Klebsiella aerogenes* KCTC 2190^T^ (CP002824)

*Raoultella ornithinolytica* JCM 6096^T^ (AJ251467)

100

99

65

62

63

68

67

**FIG S2 Neighbor-joining phylogenetic tree based on 16S rRNA gene sequences of *Klebsiella michiganensis* LDS17 and related taxa.** Bootstrap values (1000 replications) are shown as percentages at each node only if they are 60% or greater.


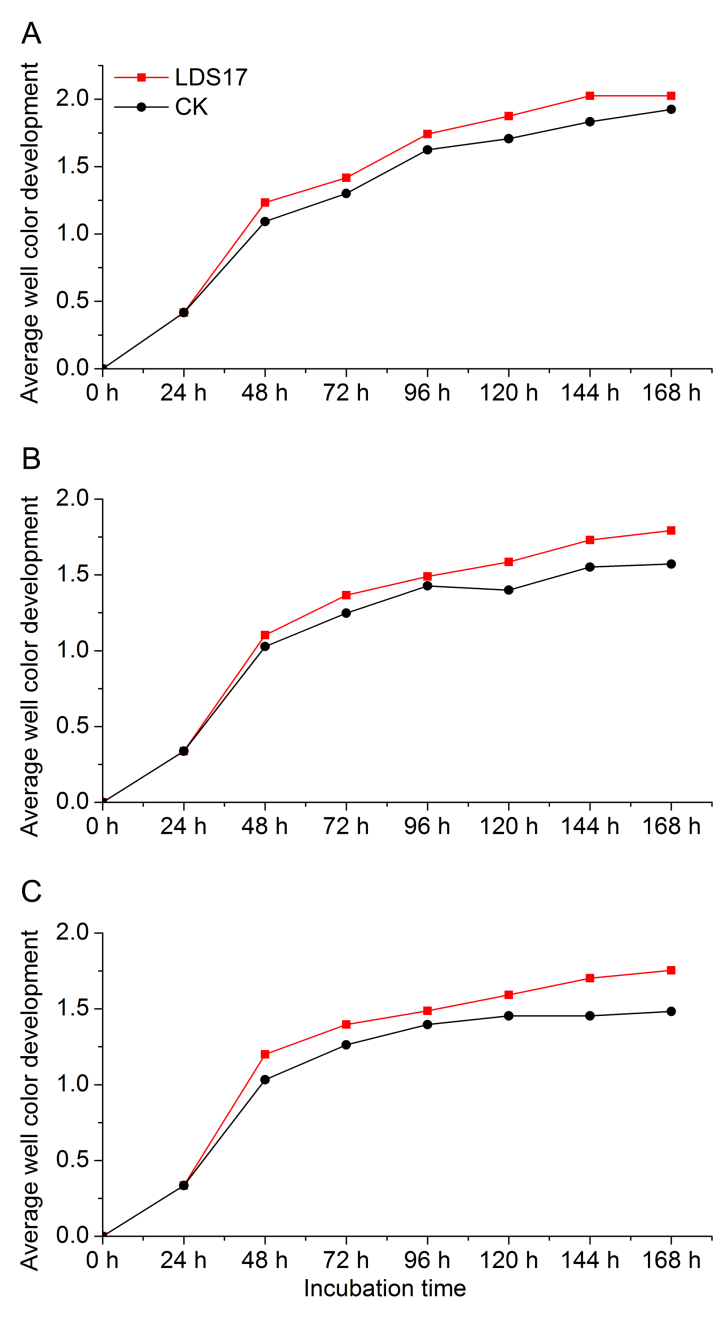


**FIG S3 Average well color development (AWCD) of metabolized substrates in the rhizosphere soil microbial communities of *Codonopsis pilosula* with *Klebsiella michiganensis* LDS17 inoculation treatment.** A. *Klebsiella michiganensis* LDS17 inoculation for 30 days. B. *Klebsiella michiganensis* LDS17 inoculation for 60 days. C. *Klebsiella michiganensis* LDS17 inoculation for 90 days.

**NesR** *Sinorhizobium meliloti* 1021 (SMc04032)

**PMI09_05533** *Rhizobium* sp. CF122

**PMI11_06415** *Rhizobium* sp. CF142

**PMI03_04239** *Rhizobium* sp. AP16

**PDO_04282** *Rhizobium* sp. PDO1-076

**KoyR** *Klebsiella michiganensis* LDS17 (I4W82_18070)

**XccR** *Xanthomonas campestris* pv. *campestris* 8004 (XC_1295)

**XagR** *Xanthomonas axonopodis* pv. *glycines* 12-2 (A9D66_16010)

**OryR** *Xanthomonas oryzae* pv. *oryzae* PXO86 (AZ54_18140)

**XocR** *Xanthomonas oryzae* pv. *oryzicola* RS105 (ACU12_06325)

**PsoR** *Pseudomonas fluorescens* Pf5 (PFL_5298)

**PipR** *Pseudomonas* sp. GM79 (PMI36_04623)

**LuxR** *Aliivibrio fischeri* ES114 (VF_A0925)

**TraR** *Agrobacterium tumefaciens* C58 (Atu6134)

100

99

98

99

100

96

85

99

100

62

50

**FIG S4 Neighbor-joining phylogenetic tree based on the amino acid sequences of LuxR family proteins.** Plant-responsive LuxR solos are highlighted in pink and quorum-sensing (QS) canonical LuxRs in purple.


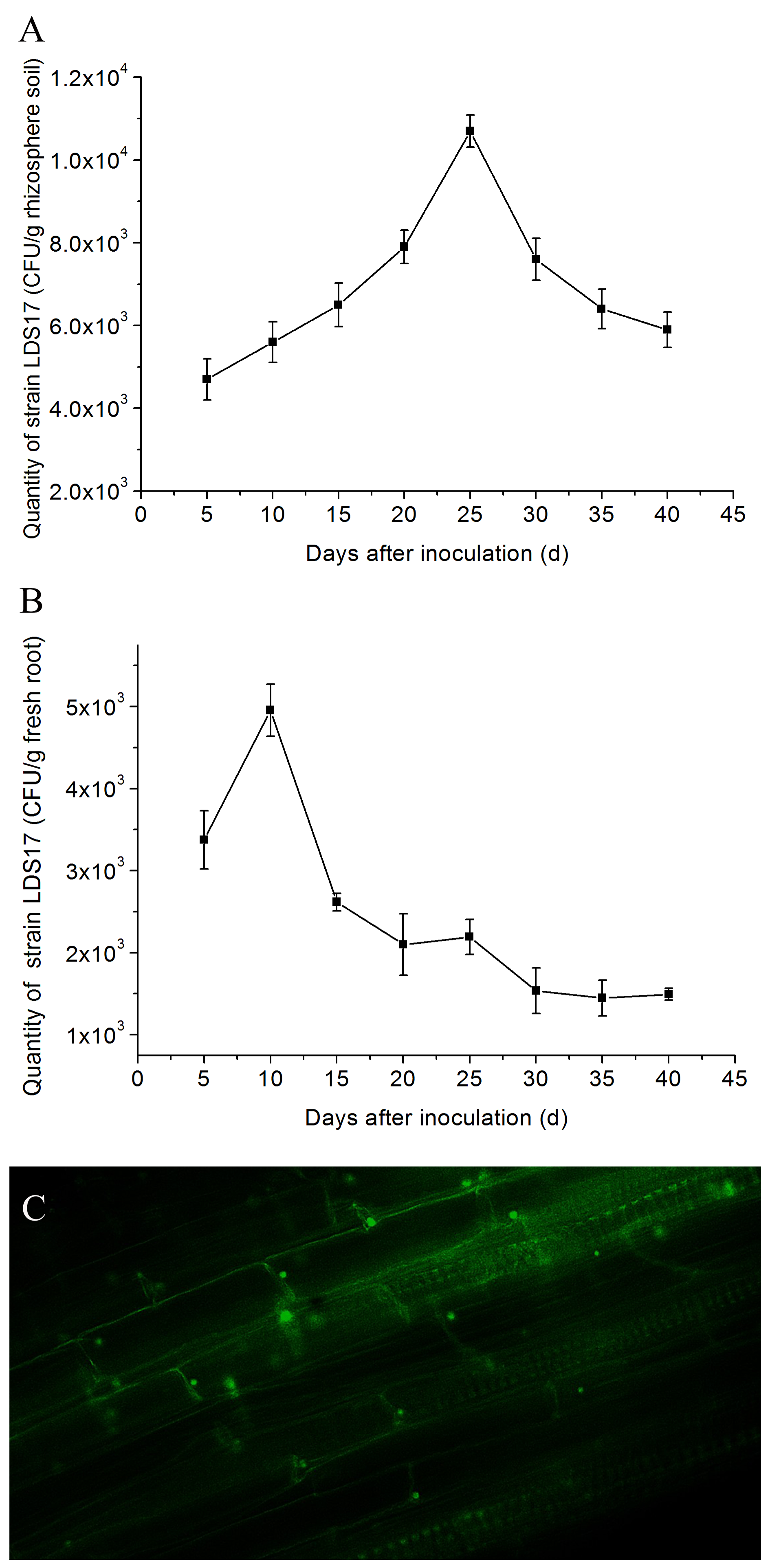


**FIG S5 Colonization of *Klebsiella michiganensis* LDS17 in the rhizosphere soil and roots of *Codonopsis pilosula* seedlings.** A, B. Colonizing populations of the GFP-labeled LDS17 strain in the rhizosphere soil (A) and roots (B) of seedlings. C. GFP-labeled LDS17 strain colonized inside and on the surface of *Codonopsis pilosula* roots.
